# A comparative evaluation of hybrid error correction methods for error-prone long reads

**DOI:** 10.1186/s13059-018-1605-z

**Published:** 2019-02-04

**Authors:** Shuhua Fu, Anqi Wang, Kin Fai Au

**Affiliations:** 1 Department of Internal Medicine, University of Iowa, Iowa City, IA 52242 USA; 2 Department of Biostatistics, University of Iowa, Iowa City, IA 52242 USA; 3 Department of Biomedical Informatics, The Ohio State University, Columbus, OH 43210 USA

## Abstract

**Background:**

Third-generation sequencing technologies have advanced the progress of the biological research by generating reads that are substantially longer than second-generation sequencing technologies. However, their notorious high error rate impedes straightforward data analysis and limits their application. A handful of error correction methods for these error-prone long reads have been developed to date. The output data quality is very important for downstream analysis, whereas computing resources could limit the utility of some computing-intense tools. There is a lack of standardized assessments for these long-read error-correction methods.

**Results:**

Here, we present a comparative performance assessment of ten state-of-the-art error-correction methods for long reads. We established a common set of benchmarks for performance assessment, including sensitivity, accuracy, output rate, alignment rate, output read length, run time, and memory usage, as well as the effects of error correction on two downstream applications of long reads: de novo assembly and resolving haplotype sequences.

**Conclusions:**

Taking into account all of these metrics, we provide a suggestive guideline for method choice based on available data size, computing resources, and individual research goals.

**Electronic supplementary material:**

The online version of this article (10.1186/s13059-018-1605-z) contains supplementary material, which is available to authorized users.

## Background

Biomedical research has been revolutionized by the advent of single-molecule long-read sequencing technologies, also termed Third Generation Sequencing (TGS) [1]. The leading platforms are Pacific Biosciences (PacBio) and Oxford Nanopore Technologies (ONT). Compared to Second Generation Sequencing (SGS) technologies that produce 100-700 bp reads, PacBio reads are on average > 10 kb, with maximum read lengths over 60 kb [1–3]. The ONT platform produces even longer reads at average of > 20 kb, with maximum read length > 800 kb [4–8]. The long read (LR) length of TGS data is particularly useful to address some of the problems that SGS short reads (SRs) fail to address due to genome complexity or combinatorial genomic events [9, 10]. For example, LRs can substantially reduce the ambiguity of read alignment at highly repetitive genomic regions and uncertainty in genome or transcriptome assembly [11]. In addition, PacBio data have been reported to exhibit fewer GC biases compared to Illumina platforms because it does not rely on PCR [12]. Moreover, the single-molecule sequencing feature of TGS allows the study of genetic variations and epigenetic modifications with single molecule resolution [1, 13, 14]. A great number of laboratories have applied TGS technologies to different research areas, including biomedical research, plant and animal sciences, and microbial genomes [15–19]. With the increasing accessibility, declining cost as well as more demonstrated utilities, the number of TGS applications is expanding exponentially (Additional file 1: Figure S1).

A caveat of the single-molecule sequencing with TGS platforms is the low signal-to-noise ratio and thereby a high error rate in base calling. The error rates of PacBio and ONT data can be up to 15% and 40% [20, 21], respectively, which are nearly two orders of magnitude greater than that of SGS technologies [22]. These high error rates pose considerable barriers for downstream data analyses, in particular for single nucleotide analyses (e.g., single nucleotide variant (SNV) calling and splice site determination) [22]. Therefore, error correction serves as a crucial step to improve data analysis and application. It is worth noting that most current TGS genome assembly tools include a built-in error-correction stage prior to assembly, which further demonstrates the importance of error correction [23, 24].

Since 2012, a number of error correction methods have been developed and generally fall into two categories: self correction and hybrid correction. The self-correction strategy corrects error-prone LRs by generating a consensus from a constructed layout of LRs. The built-in self-correction modules by PacBio and ONT output consensus of LRs from the same DNA molecule, which are termed Circular Consensus Sequencing (CCS) reads (PacBio) or 2D reads (ONT) [1, 25]. In the open-source software PBcR, the self-correction mode corrects erroneous regions using the consensus of overlapping LRs [23]. LoRMA polishes reads using long-distance dependencies from multiple alignments after employing an iterative de-Bruijn-graph-based approach with increasing *k*-mer lengths [26]. However, the self-correction strategy is not feasible for LRs with low coverage. By contrast, the hybrid-correction strategy, which utilizes high-accuracy and cost-effective SR data, can rescue more LRs, especially the low-coverage LRs. Based on algorithm design, hybrid-correction methods can be classified into three classes: alignment-based, graph-based, and dual alignment/graph-based (dual-based).

The alignment-based methods map SRs or sequences assembled from SRs to error-prone LRs and compute a consensus. pacBioToCA was the first hybrid-correction method and implemented as a part of Celera Assembler [27]. LSC increases the sensitivity of SR-LR alignment by applying homopolymer compression transformation to SRs and LRs prior to alignment [28]. Proovread performs an iterative procedure for alignment and correction with successively increasing sensitivity [29]. Nanocorr and ECTools correct LRs with SRs or SR-assembled unitigs, respectively, both of which are optimized using the longest increasing subsequence algorithm [30, 31].

The graph-based methods construct a de Bruijn graph by SRs, followed by searching for matched paths to LRs for correction. LoRDEC maps LRs to the de Bruijn graph based on shared *k*-mers [32]. Jabba seeks maximal exact matches (MEMs) between larger *k*-mers in LRs and a de Bruijn graph [33]. FMLRC builds an FM index from Burrows-Wheeler transform (BWT) of SR data and corrects erroneous regions in LRs by seeking paths in a de Bruijn graph via two passes with short and long *k*-mers respectively [34].

In addition, a few error correction tools exploit both strategies. CoLoRMap corrects LRs by finding a sequence in an overlapping graph that is constructed by mapping SRs to LRs; CoLoRMap also corrects regions without SR alignment by local assembly of unmapped SRs if they have mapped mates [35]. HALC aligns SR-assembled contigs to LRs, splits aligned contigs and the corresponding LR regions to construct a contig graph, corrects LRs by seeking paths representing alignments with minimal total edge weight, and refines repeat regions in LRs with SRs using LoRDEC [36].

Among the abovementioned methods, Jabba, ECTools, and pacBioToCA are known to discard uncorrected bases, trim, and output parts of LR data, resulting in low throughput (i.e., termed “selective methods,” underlined in the figures below).

Here, we present a comparative performance assessment of the ten available state-of-the-art error correction methods (Table 1, Additional file 2: Note 1). The objective was to establish a common set of benchmarks and to provide suggestive guideline on software choice based on data size, research interest, and computing resource requirement. The methods were evaluated in multiple dimensions, including sensitivity, accuracy, output rate, alignment rate, output read lengths, run time, and memory usage, as well as the effects of error correction on two downstream applications of LRs: de novo assembly and resolving haplotype sequences.

**Table 1:** References and software URLs of hybrid-correction methods for LRs

Error correction methods tested	Main strategy	Author, year	Version in test	Reference	Software URL
FMLRC	Graph-based	(Wang et al., 2018)	0.1.2	[34]	https://github.com/holtjma/fmlrc
Jabba	Graph-based	(Miclotte et al., 2015)	1.0.0	[33]	https://github.com/biointec/jabba
LoRDEC	Graph-based	(Salmela et al., 2014)	0.5.3	[32]	http://www.atgc-montpellier.fr/lordec/
HALC	Dual-based	(Bao et al., 2017)	1.1	[36]	https://github.com/lanl001/halc
CoLoRMap	Dual-based	(Haghshenas et al., 2016)	2016-11-30	[35]	https://github.com/sfu-compbio/colormap
ECTools	Alignment-based	(Lee et al., 2014)	2014-06-27	[30]	https://github.com/jgurtowski/ectools
LSC	Alignment-based	(Au et al., 2012)	1.beta	[28]	https://www.healthcare.uiowa.edu/labs/au/LSC/
Nanocorr	Alignment-based	(Goodwin et al., 2015)	2016-02-27	[31]	https://github.com/jgurtowski/Nanocorr
pacBioToCA	Alignment-based	(Koren et al., 2012, 2013)	From Celera Assembler version 8.1	[27]	https://sourceforge.net/projects/wgs-assembler/files/wgs-assembler/
proovread	Alignment-based	(Hackl et al., 2014)	2.14.0	[29]	https://github.com/BioInf-Wuerzburg/proovread

### Data and computing settings

We compared the performance of ten error correction methods on datasets from four model organisms with different genome sizes. Herein, we refer to the *Escherichia coli* and *Saccharomyces cerevisiae* datasets as “small” datasets that were produced by the PacBio or ONT platform, and *Drosophila melanogaster* and *Arabidopsis thaliana* datasets as “large” datasets that were produced by the PacBio platform (Table 2). To investigate the effect of SR coverage on error correction performance, we generated random subsets of each SR dataset with coverages of 5×, 20×, 50×, 75×, and 100×.

**Table 2:** Datasets used in performance assessment

Datasets	Bacteria	Yeast	Fly	Plant
Reference organism				
Name	*Escherichia coli*	*Saccharomyces cerevisiae*	*Drosophila melanogaster*	*Arabidopsis thaliana*
Strain	K-12 substr. MG1655	S288C	iso-1	Ler-0
Reference sequences	NC_000913	NC_0011{33–48} NC_001224	NT_0337{77–79} NC_0043{53–54} NC_0245{11–12} NT_037436	NC_0030{70–71} NC_0030{74–76} NC_001284 NC_000932
Genome size (Mbp)	4.64	12.13	143.73	119.67
PacBio data				
Accession number	DevNet [53]	DevNet [54]	SRX499318 [55]	SRX533607 [55]
Number of reads	55,137	220,947	6,864,972	7,515,360
Median read length	8473	5295	810	1099
Coverage	113x	112x	204x	301x
Chemistry	P6C4	P4C2	P5C3	P5C3
ONT data				
Accession number	ERR1147227, ERR1147228 [56]	ERR1883{398–402}, ERR1883389 [57]		
Number of reads	58,221	183,062		
Median read length	8652	6427		
Coverage	113x	112x		
Chemistry	R7.3	R7.3/R9		
Illumina data				
Accession number	ERR022075 [58]	SRP014568 [59]	ERX645969 [60]	SRR3166543 [45]
Number of reads	45,440,200	28,943,170	179,363,706	324,725,120
Read length	101	101/152	101	100

Note: To maximize the quality of tested LR data, CCS or 2D LR data were used if available; otherwise, subreads or template LRs from the same molecules were used instead. ONT LRs were randomly picked out to get the same data size as PacBio data. For *E. coli* data, 16.29% DNA molecules had ≥ 2 CCS passes in the PacBio dataset and 70.59% DNA molecules generated 2D LRs in ONT dataset. For *S. cerevisae* data, 0.68% DNA molecules had ≥ 2 CCS passes in the PacBio dataset and 42.38% DNA molecules generated 2D LRs in the ONT dataset. There were no CCS reads in the datasets of *D. melanogaster* and *A. thaliana*, as provided by the original authors of the resource [23]

All experiments were run on servers with 20 machines of 16 cores and 256 G memory.

### Evaluation strategies

To evaluate the performance of different error correction methods, original and corrected reads were aligned to the corresponding reference genomes with BLASR [37]. True positive (TP) positions are those with errors that are corrected by an error correction tool, whereas false negative (FN) positions are erroneous positions without correction [32]. TP and FN positions can be computed by Error Correction Evaluation Toolkit [38] by comparing original and corrected data with the reference genome. Error rates are computed as the sum of the numbers of bases of insertions, deletions, and substitutions in the alignment divided by the length of aligned regions for each read [32].

The following evaluation statistics were computed:

- *Sensitivity*: TP/(TP + FN)
- *Accuracy*: 1 − error rate
- *Output rate*: the percentage of reads output from the original data
- *Alignment rate*: the percentage of reads aligned to the reference genome relative to the output data
- *Output read length*: the lengths of output reads
- *Run time*: elapsed time consumed by the error correction tool
- *Memory usage*: peak memory taken by the error correction tool

In addition, we also examined the effects of error correction by different methods on improving de novo assembly and resolving haplotype sequences.

For error correction methods that output split reads such as Jabba, ECTools, pacBioToCA, and Nanocorr, we applied the previously published sensitivity calculation strategy [36], i.e., TH × TP/(TP + FN), where TH is the ratio of the number of output bases relative to the total number of bases from initial reads.

Error correction methods with run times greater than 20 days with twenty 16-core machines (i.e., maximal total elapsed time is ~ 3.46 × 10^7^ s) were not included in the assessment below. The alignment-based methods ECTools and LSC required run times above this limit for the large datasets, and thus their performances were not included.

## Results

Certain methods were not included in some cases for the following reasons. pacBioToCA crashed when it was applied to the *S. cerevisae* PacBio data with SR coverage of 75× or 100× and the *S. cerevisae* ONT data with SR coverage of 50× or 75×. proovread crashed when it was applied to the *S. cerevisae* PacBio data with SR coverage of 50×, 75×, or 100×. A tiny fraction (0.005%) of *S. cerevisae* ONT LRs were with length > 500 kb and were not included in evaluation by LoRDEC and HALC, owing to that both have a limit of input read length. LSC crashed on parts of *S. cerevisae* ONT data that had insufficient SR alignment, because LSC applies compression of homopolymer on SRs and LRs, while errors at homopolymer were not as frequent among ONT LRs as PacBio LRs [39]. For the large datasets, ECTools and LSC were not included due to excessive run times, Nanocorr and proovread were not included due to memory usage issues, and pacBioToCA crashed with unidentified reasons. In each result section below, we first examined the performance on PacBio datasets, followed by ONT datasets.

### Sensitivity

**Fig. 1:**
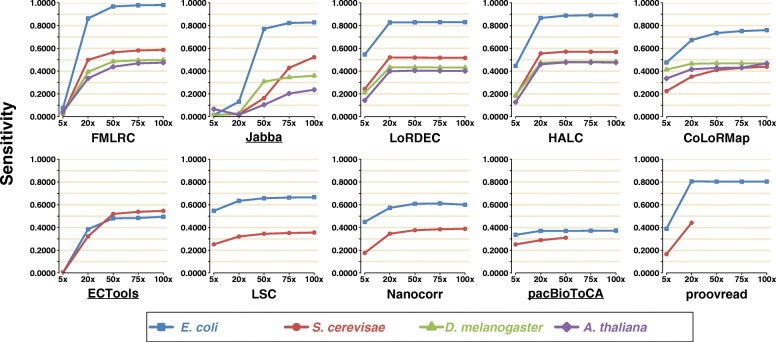
Sensitivity of ten methods on four PacBio datasets using five SR coverages. For the methods that output untrimmed LRs, sensitivity was calculated as TP/(TP + FN), where TP is true positive and FN is false negative. For the methods that output split reads, sensitivity was calculated as TH × TP/(TP + FN), where TH is the ratio of the number of output bases over the total number of bases of original reads

Sensitivity is calculated as TP/(TP + FN), with TP being the number of erroneous positions that are corrected by error correction tool, and FN being the number of erroneous positions without correction (see details in “Evaluation strategies”). In general, most methods demonstrated increased sensitivity on correcting PacBio LRs with increasing SR coverage (Fig. 1, Additional file 3: Table S1a). Specifically, FMLRC, Jabba, LoRDEC, HALC, CoLoRMap, ECTools, and proovread improved dramatically with increasing SR coverage until they reached saturation (defined as a sensitivity gain of ≤ 0.03 improvement). By contrast, LSC, Nanocorr, and pacBioToCA only demonstrated a mild increase in sensitivity with SR coverage from 5× to 20×. This is likely because LSC, Nanocorr, and pacBioToCA can correct LR regions with low SR coverage after SR-LR alignment, while the methods utilizing a graph-based strategy (FMLRC, Jabba, LoRDEC, HALC, and CoLoRMap) require sufficient SR coverage to build the graph.

LoRDEC, HALC, and proovread reached sensitivity saturation at 20× SR coverage, while FMLRC and ECTools were at 50×. Depending on the LR dataset, CoLoRMap and Jabba saturated at high SR coverages of 20–50× and 75–100×, respectively (and beyond 100× for Jabba on the *S. cerevisae* and *A. thaliana* datasets). In terms of the saturation sensitivities, FMLRC dominated the other tools on all LR datasets (e.g., for *E. coli* 0.9808 vs. 0.3719–0.8897 and except *A. thaliana* dataset). However, when only 5× SR coverage was available, LoRDEC, HALC, CoLoRMap, LSC, Nanocorr, pacBioToCA, and proovread provided a higher sensitivity than FMLRC on the small datasets (0.1651–0.5458 vs. 0.0242–0.0749). pacBioToCA generally had very low or the lowest sensitivity in most cases (0.2507–0.3719).

Overall, the sensitivities achieved with the *E. coli* PacBio dataset (i.e., the smallest LR dataset) by most tested methods were generally greater than those on other datasets, with the exception of ECTools, which exhibited a slight higher saturation sensitivity with the *S. cerevisae* dataset (0.5458 vs. 0.4936). Jabba, ECTools, and pacBioToCA processed and output incomplete data, which may contribute to their lower sensitivities (0.0024–0.8326 on the small datasets).

With respect to SR coverage, the peak sensitivities of correcting ONT data generally were lower compared to PacBio data with the same size of LRs, with the exception of FMLRC, LoRDEC, HALC, and proovread (Additional file 1: Figure S2, Additional file 3: Table S1b). Similar with the results of the PacBio data, the sensitivities for *E. coli* ONT dataset were mostly higher than those for *S. cerevisae* ONT dataset, with the exception of ECTools (e.g., 0.3502 vs. 0.3896 with 100× SRs).

### Accuracy

**Fig. 2:**
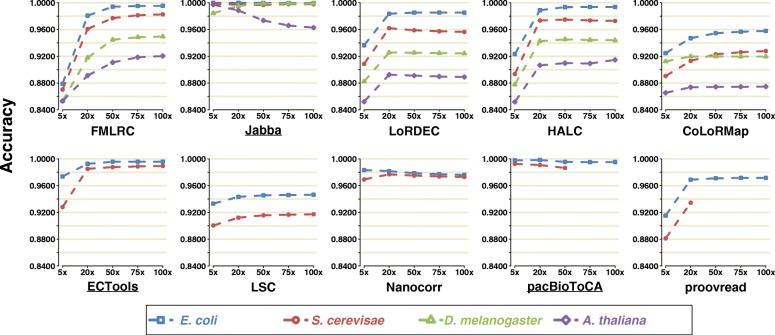
Accuracy of ten methods on four PacBio datasets using five SR coverages. Accuracy was defined as 1 − error rate, where error rate was calculated as the total number of bases of insertions, deletions, and substitutions in the alignment divided by the total aligned length of the corresponding genome

We next assessed the accuracy of the corrected PacBio LRs, where accuracy was computed as 1 − error rate (i.e., 1 − sum of base numbers of insertions, deletions, and substitutions in the alignment divided by the length of aligned region for each read; see details in “Evaluation strategies”). For all methods except Jabba, Nanocorr, and pacBioToCA, accuracy increased with increasing SR coverage, and the accuracy patterns with respect to SR coverages were similar to the sensitivity results for each method (Fig. 1 and Fig. 2, Additional file 4: Table S2a). Since Jabba outputs a selected proportion of LRs, the accuracy was the highest for all datasets and at all SR coverages including shallow 5× (e.g., ≥ 0.9996 and ≥ 0.9967 for *E. coli* and *S. cerevisae* datasets, respectively); this level of accuracy approaches the quality of Illumina data. Therefore, there is little additional benefit by increasing SR coverage for the accuracy of Jabba, though it is important to note that sensitivity of Jabba was improved with more SRs. For the same reason, pacBioToCA was also of high accuracy (≥ 0.9953 and ≥ 0.9866 for *E. coli* and *S. cerevisae* datasets, respectively). However, it is interesting that the other selective method ECTools had relatively low accuracy at 5× SR coverage accuracy (0.9738 and 0.9281 for *E. coli* and *S. cerevisae* datasets, respectively), though the accuracy at ≥ 20× SR coverage was similar to Jabba. Altogether, these selective methods output high-accuracy LR fragments under sufficient SR coverage, although they fail to output whole datasets of full-length LRs. The trend and accuracy at saturating coverage levels with FMLRC were very similar to ECTools, though the relative accuracy of FMLRC at 5× SR coverage was far lower (< 0.88). LoRDEC, HALC, and Nanocorr comprised the intermediate group, with accuracy saturation ranging from 0.9619 to 0.9887 on the small datasets. CoLoRMap, LSC and proovread underperformed comparatively with accuracy saturations ranging from 0.9122 to 0.9718 on the small datasets.

It is worth noting that both sensitivity and accuracy reach to the saturated values at the same SR coverage for all methods except Jabba, ECTools, and Nanocorr. That is, it is possible to achieve the best sensitivity and accuracy simultaneously as long as sufficient SRs are provided.

For the non-alignment-based methods that can be applied to all four PacBio datasets, they generally achieved greater or equivalent accuracies on the small datasets as compared to the large datasets. For unknown reasons, increasing SR coverage reduced the accuracy of Jabba with the *A. thaliana* dataset.

Prior to correction, raw ONT LRs had lower accuracy compared to PacBio raw LRs (0.8221 vs. 0.8677 on *E. coli* data and 0.7959 vs. 0.8501 on *S. cerevisae* data), and such disadvantage of ONT data remained after error correction (except correction by the selective methods) (Additional file 1: Figure S3, Additional file 4: Table S2b). The output PacBio and ONT LRs by the selective methods had no substantial difference of accuracy on the small datasets, as they only trim and output high-quality fragments of LRs (0.9281–0.9999 vs. 0.9082–0.9999). Considering the other methods, the accuracy difference between corrected ONT and PacBio LRs decreased, with exception of LSC.

### Output rate

**Fig. 3:**
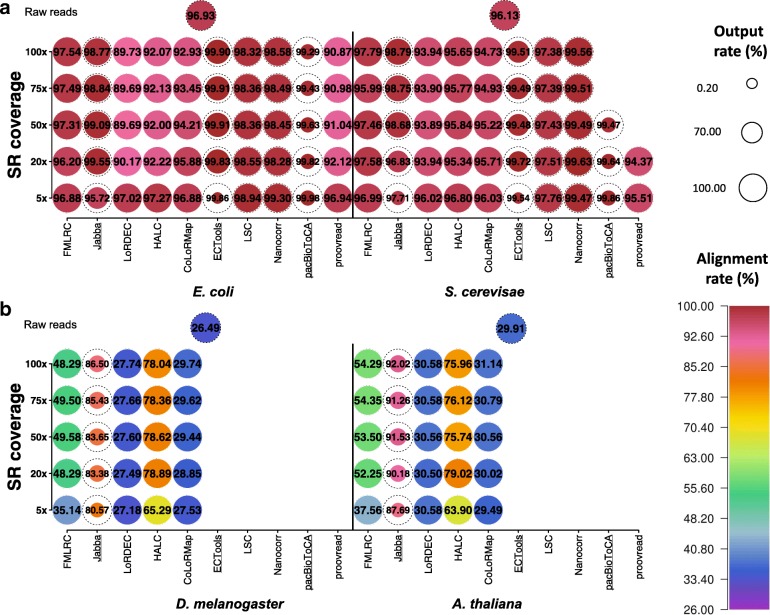
Output rates and alignment rates of ten methods on PacBio datasets using five SR coverages. The comparisons were performed on **a** the small datasets (i.e., *E. coli* and *S. cerevisae*) and **b** the large datasets (i.e., *D. melanogaster* and *A. thaliana*). The tests that failed to complete are not shown. The circle diameter is positively correlated to the output rate, with the scale on the right side ranging from 0.20 to 100.00%. The dashed circles represent 100.00% output rates. The numbers labeled in circles represent the corresponding alignment rates that are also scaled with rainbow colors. The output rates of raw reads are 100.00% and the corresponding alignment rates are also shown for comparison

Errors are distributed randomly among LRs, and some LRs are so error-prone that they are hard to correct and thus may not be output by the error correction software [1]. Therefore, it is important to evaluate the amount of data remaining after error correction. When PacBio datasets were corrected, all methods, except for Jabba, ECTools, and pacBioToCA, output all or > 90% of input data (Fig. 3, Additional file 1: Figure S4a, Additional file 5: Table S3a). Specifically, FMLRC, HALC, and CoLoRMap output the whole dataset. LoRDEC and proovread only excluded a trivial number of reads (output rate: > 99.99% and ≥ 99.16%, respectively). LSC and Nanocorr lost a small amount of data (output rate: 94.17–97.16% and 92.84–95.70%, respectively) since these alignment-based methods may not be able to correct and thereby would not output LRs with no aligned SRs. In particular, the output rate with these methods did not depend on SR coverage and LR data size. Note that the output rate could be increased to 100% by changing the software design and implementation to include both corrected and uncorrected LRs. A high output rate can allow users to maintain data for different research interests and execute different data analysis strategies.

In contrast, the selective methods Jabba, ECTools, and pacBioToCA output only the trimmed reads with corrected regions, so their output rates were far lower than 100%. With an output rate of ~ 21% regardless of SR coverage, pacBioToCA generally had the lowest output rate among all tests and methods. Exceptions to this trend include Jabba at 5× SR coverage for all LR datasets (15.50–18.91%) except *E. coli*, and ECTools at 5× SR coverage for the small datasets (0.40–1.28%). With Jabba and ECTools, the output rate increased considerably between 5× and 20× SR coverage and continued to increase until saturation at 50–75× and 20–50× SR coverage, respectively. However, these two methods still lost a significant amount of data even at saturation (e.g., output rate ~ 32% for Jabba on the large datasets).

When ONT datasets were corrected, FMLRC and CoLoRMap also output whole datasets, and the output rate of LoRDEC, HALC, and proovread was very high as well (≥ 97.77%) (Additional file 1: Figure S4b and Figure S5, Additional file 5: Table S3b). It should be noted that LoRDEC and HALC that uses LoRDEC as a module failed to correct LRs with lengths > 500 kb and therefore excluded a small portion of LRs on *S. cerevisae* data. Except these high-output-rate methods, the other methods had lower output rates for ONT datasets than the corresponding PacBio datasets from the same species (0.22–77.54% vs. 0.40–97.16%), as higher error rate of raw data resulted in fewer correctable LRs.

### Alignment rate

Most sequencing data analysis starts with alignment, especially to reference genomes, so a high alignment rate is critical for subsequent analyses [40]. While accuracy can affect alignment rate, these two metrics should be evaluated separately since the alignment tools (e.g., BLASR) can tolerate certain numbers of errors. More importantly, accuracy is critical for nucleotide analysis, such as variant calling and alternative splice site detection, while some other applications such as fusion gene detection and abundance estimation require high alignment rate but not high resolution at the single nucleotide level [41]. For the small datasets from the PacBio platform, the alignment rates were almost contrastive to the output rates: ECTools and pacBioToCA had the highest alignment rates (≥ 99.48% and ≥ 99.29%, respectively) though they had the poorest output rates; LSC and Nanocorr had intermediate alignment rates (≥ 97.38% and ≥ 98.28%, respectively), similar to their rank for output rate; FMLRC, LoRDEC, HALC, CoLoRMap, and proovread, which output all or almost all LRs, had the lowest alignment rates (95.51–97.27% with 5× SR coverage) (Fig. 3, Additional file 1: Figure S4a and Figure S6a, Additional file 6: Table S4a). It is likely that Jabba, ECTools, and pacBioToCA selectively output high-quality LR fragments that were easy to align, while the high output-rate methods included uncorrected LRs that thus caused lower alignment rate. Overall, the alignment-based methods (except proovread) tended to achieve higher alignment rates because their output LRs had been corrected by aligned SRs, which in turn could serve as the seeds to align the output LRs.

As SR coverage increased from 5× to 100×, the alignment rates of LoRDEC, HALC, CoLoRMap, and proovread all decreased by 1–7% with the small datasets. A similar yet smaller decrease (< 1%) in alignment rate with respect to SR coverage existed in LSC, Nanocorr, and pacBioToCA with the small datasets. As exceptions, the alignment rates of FMLRC, Jabba, and ECTools did not have a clear dependence on SR coverage.

The alignment rates for the graph-based methods were much lower for the large datasets as compared to the small datasets (Additional file 1: Figure S6b). Jabba achieved the highest alignment rates with the large datasets. As SR coverage increased, the alignment rates of LoRDEC and CoLoRMap increased mildly but were still at low values of ~ 30%. In contrast, the alignment rates for FMLRC, Jabba, and HALC improved strikingly with increasing SR coverage, where Jabba was still highest at 86.50% and 92.02% with *D. melanogaster* and *A. thaliana* datasets, respectively.

Because of the lower accuracy (Additional file 4: Table S2b), raw ONT LRs had lower alignment rates than PacBio raw LRs (74.02% vs. 96.93% on *E. coli* data and 57.64% vs. 96.13% on *S. cerevisae* data; see Additional file 6: Table S4b). After error correction, the output of most methods had much lower alignment rates of ONT data than the corresponding PacBio data from the same species (e.g., 74.30–82.64% vs. 96.20–97.54% on *E. coli* data by FMLRC), with the exception of the selective methods and Nanocorr that merely output parts of datasets (Additional file 1: Figure S5 and Figure S6c).

### Output read length

**Fig. 4:**
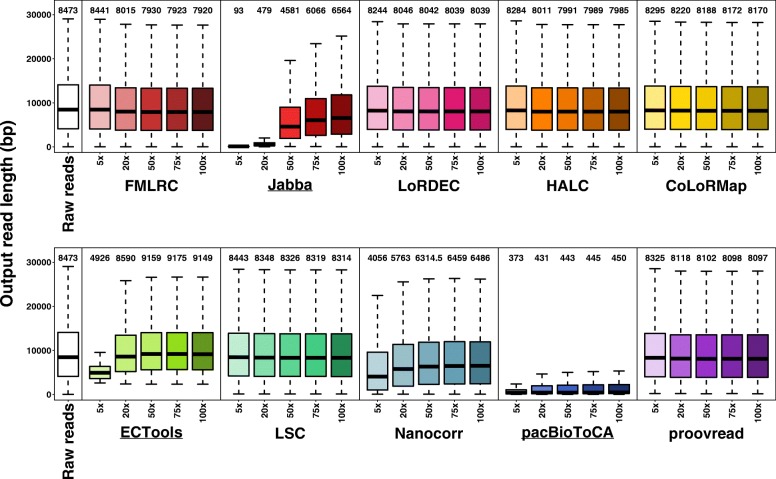
Output LR lengths of ten methods on *E. coli* PacBio dataset using five SR coverages. The length distributions were shown in boxplots (outliers not shown). The medians are labeled above the corresponding boxplots

LR length is the main advantage of TGS data for many applications (e.g., genome assembly) [31]. Thus, we investigated how well the methods maintain the read lengths on PacBio datasets (Fig. 4, Additional file 1: Figure S7). All methods, except for the three selective methods and Nanocorr, output read lengths that were similar to the length of the input data (median length: 7920–8443 bp output vs. 8473 bp input for *E. coli* dataset and 5006-5453.5 bp vs. 5295 bp for *S. cerevisae* dataset). The output read lengths generally mildly decreased as SR coverage increased since insertions are the main errors in PacBio data, i.e., the increasing SR coverage improved the accuracy, and the corrected reads with fewer insertion errors were slightly shorter [12]. In contrast, the selective methods output very distinct length profiles that were much longer or much shorter than input LRs, which occurred due to two reasons: (1) the selective methods merely output a small proportion of LRs and (2) they output trimmed reads with corrected regions. For the small datasets, Jabba output very short reads (median length: 93 bp for *E. coli* dataset and 360 bp for *S. cerevisae* dataset) at 5× SR coverage and generally exhibited significant increases in output read lengths with increasing SR coverage, which was consistent with the improved output rates (up to > 80%). Although the median output read length of ECTools was far shorter than the median input length (4926 bp vs. 8473 bp for *E. coli* dataset and 3627 bp vs. 5295 bp for *S. cerevisae* dataset) at 5× SR coverage, it significantly increased as SR coverage was increased from 5× to 50× and then saturated. The corresponding median read length at 50× SR coverage was much longer than the input read length (9159 bp for *E. coli* dataset and 6403 bp for *S. cerevisae* dataset). In case of sufficient SR coverage, ECTools appeared to selectively remove some short LRs for an unknown reason. The median lengths of output reads by pacBioToCA were only 211–450 bp, and no substantial improvement was observed with increasing SR coverage. pacBioToCA appeared to trim LRs into many small corrected pieces. Interestingly, Nanocorr also output shorter reads (longest median length: 6486 bp vs. 8473 bp for *E. coli* datasets and 3278 bp vs. 5295 bp for *S. cerevisae* dataset), and read length increased as SR coverage increased, similar to Jabba. However, Nanocorr output read length was generally longer than Jabba, especially for shallow SR coverage (i.e., 5×). The output rate of Nanocorr was still around 95% as compared to the selective methods of 0.40–88.35% (Fig. 3). A possible explanation is that Nanocorr trimmed small fragments from each LR, such as the 5′ and 3′ termini where SR coverage is missing, the selective methods trimmed single reads into multiple corrected fragments. The remaining methods output intact reads including corrected and uncorrected regions.

As SR coverage increased, there was a mild trend of decreasing output ONT read length as *E. coli* PacBio dataset, by the methods except the selective ones, LSC and Nanocorr (Additional file 1: Figure S8). The overall output read lengths by LSC were much longer than the raw ONT LRs (median length: 9237–9263 bp vs. 8652 bp on *E. coli* ONT data and 9367.5–9626 bp vs. 6427 bp on *S. cerevisae* ONT data), as longer ONT LRs had higher probability to have SR alignment for correction by LSC.

### Run time

**Fig. 5:**
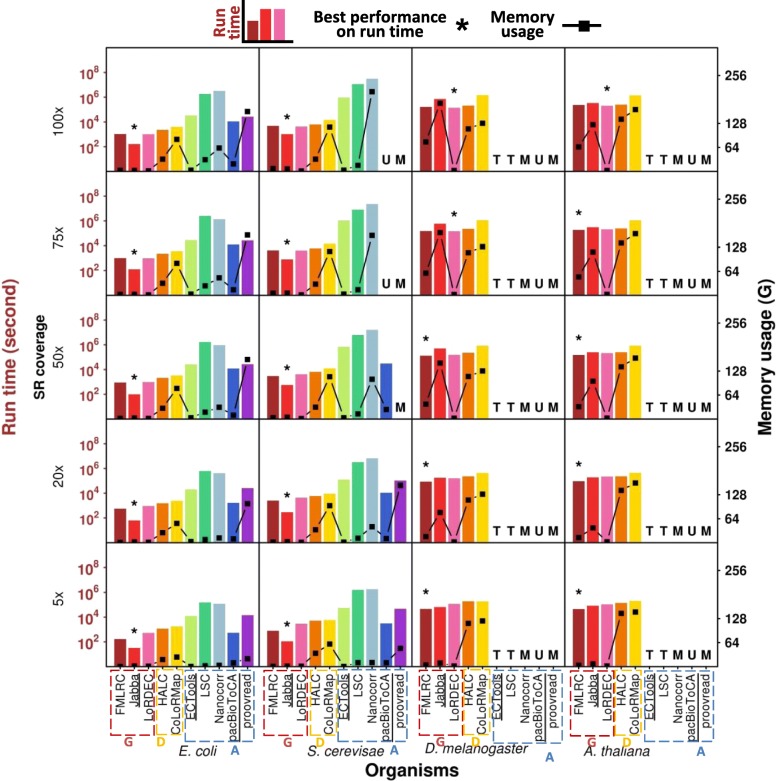
Run time and memory usage of ten methods on PacBio datasets using five SR coverages. The comparison was performed on four PacBio datasets with five SR coverages. The performances for run times are shown with bars and for memory usages are shown with line plots. The best performance of run time is labeled with an asterisk. Methods are organized from left-to-right as follows: “G”- graph-based (FMLRC, Jabba and LoRDEC), “D”- dual-based (HALC and CoLoRMap), and “A” - alignment-based (ECTools, LSC, Nanocorr, pacBioToCA and proovread). “T/M/U” - the method failed due to ***T***ime/***M***emory/***U***nidentified issue

Run time is an important practical factor users need to consider before they apply an error correction method. For PacBio LRs, the graph-based methods generally processed the small datasets with much shorter run time relative to the alignment-based methods (magnitude of 10–10^3^ s vs. magnitude of 10^2^–10^7^ s) (Fig. 5, Additional file 1: Figure S9a, Additional file 7: Table S5a). The differences were magnified with increasing SR coverage, i.e., the run times for the alignment-based methods were more dependent on SR coverage than the graph-based methods. The graph-based methods use SR-constructed graphs rather than direct usage of SRs in correction. Graph size may not considerably enhance when SR data are sufficient, and also the graph construction stage is very time-efficient [42, 43]. SR coverage effect thereby is not as striking as the alignment-based methods on run time. Jabba outperformed all other methods on the small datasets with different SR coverage in terms of run time (only 32–1041 s), while FMLRC or LoRDEC had the shortest run times on the large datasets (44,312–202,940 s). Jabba has been previously reported to use long run time on the large datasets [36]. As expected, the run times for the dual-based methods, HALC and CoLoRMap, were generally intermediate (e.g., magnitude of 10^3^–10^4^ s for the small datasets) between the graph-based and the alignment-based methods. Among the alignment-based methods, pacBioToCA required the shortest run time (e.g., 534–29,956 s for the small datasets), though it crashed due to unidentified reasons when the input was higher coverage SR data (i.e., 75× and 100× for *S. cerevisae* data) or the large datasets. The alignment-based methods ECTools, LSC, Nanocorr, and proovread had much longer run times (magnitude of 10^4^–10^7^ s) with the small datasets as compared to other methods. Among the slowest methods, input LRs were divided into small portions to run ECTools, LSC, and Nanocorr as the software manuals suggested. This protocol of splitting LRs for SR alignment separately has been reported to take longer total run time [44], so the run times of these methods may be overestimated. In particular, ECTools and LSC did not complete the large datasets by the 20-day run time limit; Nanocorr and proovread exceeded the memory usage limit of 256 G and crashed with higher SR coverage or the large datasets.

ONT LRs with the same data size as PacBio data generally took less run time, e.g., the run time of *E. coli* ONT and PacBio data by FMLRC was 119–944 s and 169–1072 s, respectively (Additional file 1: Figure S9b and Figure S10, Additional file 7: Table S5b). Compared to PacBio data, ONT raw reads are of higher error rate and thus have more extremely erroneous regions [1] that are not correctable. Few SR-LR alignments or paths between solid *k-mers* can be found at extremely erroneous regions and thus correction by the alignment-based methods or the graph-based methods was difficult. With the exception of the selective methods, the graph-based methods still outperformed the alignment-based methods on the ONT data (magnitude of 10^2^–10^4^ s vs. magnitude of 10^4^–10^7^ s), and the dual-based methods were between them (magnitude of 10^3^–10^4^ s). This trend was the same as the PacBio data.

### Memory usage

Memory usage is another factor that users should consider with their computing resources available. In terms of memory usage for correcting PacBio LRs, LoRDEC was one of most efficient methods with no appreciable SR coverage dependence (Fig. 5, Additional file 1: Figure S11a, Additional file 8: Table S6a), which only required 1–2 G on both the small and large datasets. This is likely because LoRDEC utilizes GATB core to construct de Bruijn graph from SRs and traverse paths in the graph, which is particularly memory-efficient [43]. FMLRC and Jabba had similar memory efficiencies and very mild SR coverage dependence for the small datasets (from 1 to 8 G). By contrast, the memory usage for Jabba, and to a lesser extent FMLRC, exhibited substantial SR coverage dependence for the large datasets, e.g., Jabba’s memory usage for *D. melanogaster* data with 100× SR coverage exceeded 180 G, majorly given to the storage of the enhanced sparse suffix array [33].

The dual-based methods HALC and CoLoRMap generally were less memory-efficient than the graph-based methods (e.g., 19–118 G vs. 1–8 G on the small datasets) and some alignment-based methods (i.e., ECTools, LSC, and pacBioToCA for the small datasets (0.32–31 G)). Memory usage of HALC was relatively stable for LR datasets regardless of SR coverage (19–36 G on the small datasets and 114–142 G on the large datasets). The memory usage for CoLoRMap for the small datasets rose as SR coverage increased and reached a plateau at 20× or 50× SR coverage (in the neighborhood of 82 G and 113 G for *E. coli* and *S. cerevisae* datasets, respectively), while it was of little dependence on SR coverage and maintained at high memory usage (122–165 G) for the large datasets.

Among the alignment-based methods, ECTools, LSC, and pacBioToCA were very memory-efficient. In particular, ECTools used the similar memory (≤ 4 G) as LoRDEC for the small datasets. However, two other alignment-based methods, Nanocorr and proovread, required a lot more memory (up to > 200 G for the small datasets) and their memory usage highly depended on LR data size or SR coverage. They crashed due to > 256 G memory usage with the large datasets; proovread also crashed with the *S. cerevisae* LR datasets at ≥ 50× SR coverage.

Considering practical application, regular desktops with 32 G memory could be sufficient to run FMLRC, Jabba, LoRDEC, ECTools, and LSC for the small datasets. By contrast, CoLoRMap, Nanocorr, and proovread required far more memory as SR coverage increased and High-Performance Computing (HPC) machines should be used. To run the large datasets, except that LoRDEC required only 2 G memory, HPC machines were required for FMLRC, Jabba, HALC, and CoLoRMap (e.g., some cases even reached > 128 G memory usages).

Overall, ONT LRs had less memory usage than PacBio LRs (e.g., 5–33 G vs. 21–161 G on the small datasets by proovread) (Additional file 1: Figure S10 and S11b, Additional file 8: Table S6b). The memory consuming method proovread did not crash on ONT data as PacBio data, perhaps because fewer SR-LR alignments could be obtained due to the lower accuracy of raw ONT LRs [1]. The graph-based methods were also memory efficient on ONT LRs (0.15–2 G for the small datasets).

### Overall performance

**Fig. 6:**
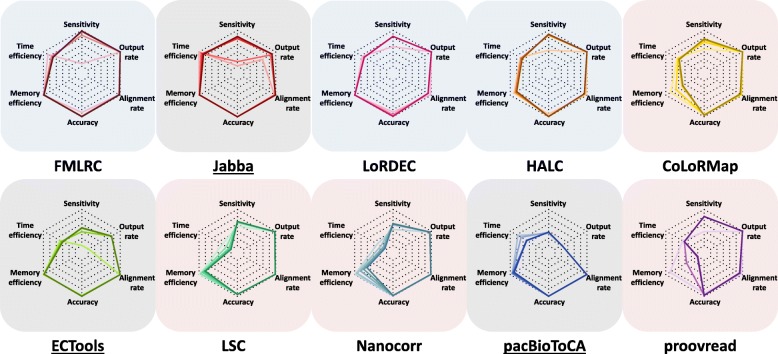
Radar charts of ten methods on *E. coli* PacBio dataset using five SR coverages. The six performance metrics include sensitivity, time efficiency, memory efficiency, accuracy, alignment rate, and output rate. The color depth is positively correlated to SR coverage. Time efficiency is computed as the log10 transformed run time that is further normalized to 0–1 range: 0 corresponds to the longest run time and 1 corresponds to the shortest run time among ten methods with five SR coverages. Memory efficiency is computed as the memory usage that is normalized to 0–1 range: 0 corresponds to the highest memory usage and 1 corresponds to the lowest memory usage among ten methods with five SR coverages. According to the overall performance, ten methods are grouped and shaded: blue—the graph-based FMLRC and LoRDEC plus the dual-based HALC; pink—the alignment-based LSC, Nanocorr, and proovread plus the dual-based CoLoRMap; gray—the selective methods Jabba, ECTools, and pacBioToCA

We here assessed the overall performance of the ten error correction methods by taking into consideration the metrics evaluated above. Using the *E. coli* PacBio dataset as an example, the methods were segregated into three groups: graph-/dual-based, alignment-/dual-based, and selective methods (Fig. 6). Generally, the graph-/dual-based methods had better overall performance, and FMLRC had the best overall performance.

The graph-based methods FMLRC and LoRDEC together with the dual-based method HALC had very similar performances: superior sensitivity and accuracy, high output rate and alignment rate, and efficient memory usage (shaded in blue in Fig. 6). Their time efficiency was better than the others yet lower than Jabba, though Jabba only output part of the data. The only caveat was that sensitivity relied on SR coverage: their sensitivities were poor on 5× SR coverage data, whereas 20× SR coverage was sufficient to achieve reasonable performance. In particular, FMLRC provided the highest sensitivity at 50× SR coverage. HALC required more memory usage as it is dual-based, and the alignment part may not be memory-efficient.

The second group consisted of the alignment-based methods LSC, Nanocorr, and proovread and the dual-based method CoLoRMap (shaded in pink in Fig. 6). As compared to the first group, these tools had comparable yet slightly lower sensitivities, accuracies, and output rates. However, their alignment rates were generally slightly higher, especially compared to LoRDEC and HALC (90.87–99.30% vs. 89.69–97.27%). The second group shared the drawback of poor computing efficiency. Except for LSC, the methods in this group required high memory usage that increased dramatically with SR coverage. Because of the memory issue, Nanocorr and proovread failed in the large datasets. In addition, they all had exceptionally long run times. The run times for LSC (from 149,736 s to 3,266,168 s) and Nanocorr (from 120,518 s to 1,816,642 s) increased by one order of magnitude as SR coverage increased from 5× to 100×. Within this group, a specific weakness of Nanocorr was the relatively short output read length (Fig. 4) (range of median lengths: 4056–6486 bp for Nanocorr vs. 8097–8443 bp for the other members).

Output read length and output rate were notable issues for the selective methods (shaded in gray in Fig. 6). Because these methods output a small subset of the data (i.e., very low output rate and sensitivity), they were memory efficient (0.35–20 G) and their output data were of high accuracy (0.9738–0.9999) and alignment rate (95.72–99.98%). However, run times were still long except Jabba (534–33,257 s vs. 32–161 s). It is worth noting that Jabba rescued the output rate and sensitivity with 75× SR coverage to approach the output rate and sensitivity of the other two groups of methods. While the increasing SR coverage improved the output rate of ECTools (from 1.28 to 72.95%), it could not improve the output rate of pacBioToCA (from 21.53 to 21.63%) but extended the run time (from 534 to 11,142 s).

As compared to the *E. coli* dataset, the sensitivity and alignment rate decreased when larger PacBio LR datasets were applied (Additional file 1: Figure S12). The overall performance of the methods was very similar between the two small datasets. For the large datasets, the graph-based and the dual-based methods ran successfully while the alignment-based methods failed. In particular, the first group mentioned above maintained high accuracy and output rate. Although the overall performance of all methods decreased for the large datasets, several graph-/dual-based methods still stood out on specific metrics, such as a high computing efficiency by LoRDEC and a relatively high alignment rate by Jabba and HALC.

Overall, high computing efficiency was a remarkable advantage for the graph-based methods over the alignment-based methods, which is very likely due to the algorithm design [34]. On the other hand, the alignment-based methods could achieve reasonable performance with shallow SR coverage. The dual-based method HALC uses LoRDEC as a plugin and thus had similar performance to the graph-based methods, while the other dual-based method CoLoRMap is similar to the alignment-based methods.

Most methods had similar performance on correcting ONT data with PacBio data, yet with lower alignment rate and accuracy (Additional file 1: Figure S13). Raw ONT LRs had lower accuracy, resulting in bigger challenges of correction. We next analyzed the effects of error correction on two downstream applications: de novo assembly and resolving haplotype sequences.

### Performance on improving de novo assembly

An important goal of error correction is to provide better-quality LRs for de novo assembly, so we also evaluated the error correction performance by ten error correction methods on improving de novo assembly (Additional file 2: Note 2, Additional file 9: Table S7). All assemblies were performed by Miniasm [45], a software that performed assembly without built-in error correction or consensus calling. There were some failures of assembly due to insufficient genome coverage of corrected LRs, including those by the selective methods, and LSC that crashed and only output parts of *S. cerevisae* ONT dataset (labeled with “NA” in Additional file 9: Table S7).

First, we assessed the number of assembled contigs that should be ideally close or equal to the number of chromosomes. When assembling *E. coli* genome by PacBio data, both raw and corrected LRs (except by the selective methods) generated similar numbers of contigs (1–3) with the number of chromosome (1). However, the assemblies of more complicated genomes (i.e., *D. melanogaster* and *A. thaliana*) by corrected LRs produced a lot more contigs than the raw data, with an exception of the selective method Jabba. The contig numbers assembled from ONT LRs were closed to those from the corresponding PacBio datasets.

N50 of the assemblies by selective-method-corrected LRs was far shorter than the others, including the ones by raw data, because the selective methods only output small fractions of LRs. Considering the other methods, the assemblies of the simple *E. coli* genome by corrected LRs produced N50 closer to the true value, especially when enough SR coverage was provided (e.g., 4.64 Mb by FMLRC-corrected PacBio LRs with 75× SRs vs. 4.64 Mb *E. coli* genome size). When assembling the more complicated genome of *S. cerevisae*, N50 improved significantly with corrected LRs except by the selective methods, compared to raw reads (e.g., 666,643–817,703 bp using 100× SRs vs. 586,605 bp by raw reads). Interestingly, the corrected LRs mostly provided shorter N50 for the large datasets than raw LRs (e.g., 13,830–1,331,739 bp vs. 1,501,783 bp on *A. thaliana* data), given that hybrid correction is harder for complex genomes and this process may introduce errors, resulting in misassemblies. Compared to PacBio LRs, the maximal N50 of assemblies by the corresponding ONT LRs (with/without correction) was shorter (e.g., 817,703 bp vs. 782,610 bp on *S. cerevisae* data).

We next examined genome fraction that reflects completeness of assemblies. The assemblies on the PacBio small datasets by the methods (including FMLRC, HALC, and CoLoRMap) with 100% output rate (Additional file 5: Table S3a) had genome fractions closed or equal to 1. Although ECTools and pacBioToCA only output LR fragments selectively, they still achieved genome fractions closed or equal to 1 with sufficient SR coverage (e.g., ≥ 50×). However, genome fraction by Jabba was low even using 100× SRs (0.82 on *E. coli* data and 0.51 on *S. cerevisae* data), albeit it had higher output rates than the other two selective methods (15.50–88.35% vs. 0.40–73.00% on small PacBio datasets, Additional file 5: Table S3a). Both raw and corrected LRs of the large datasets (except being corrected by Jabba) had higher genome fractions than the small datasets (1.07–1.82 vs. 0.97–1.05), while those by Jabba remained very low (≤ 0.39). Assemblies on ONT LRs with correction generally had genome fractions lower than those by PacBio LRs (e.g., 0.93–0.98 vs. 1.01 on *S. cerevisae* data by FMLRC).

The effect of ten hybrid-correction methods on contig sequence accuracy (i.e., 1 − error rate of contig sequences) was also evaluated. With hybrid correction prior to assembly, contig sequence accuracy was improved dramatically for all methods (e.g., for *E. coli* PacBio data, assembly by raw data 0.8686 vs. corrected data 0.8828–0.9995, more details in Additional file 9: Table S7a). The improvement generally increased with SR coverage and tiny variance existed after saturation. Although the assembly by Jabba-corrected data provided the highest contig sequence accuracy in most cases, it should be noted that the corresponding genome fraction was very low.

Altogether, except the selective methods, assemblies by the other hybrid-corrected LRs improved contig numbers, N50, and genome fraction in most cases. Error correction methods except selective ones generally improved contig sequence accuracy that increased with SR coverage. The selective methods, especially Jabba, helped to achieved assemblies with highest contig sequence accuracy, while they sacrificed the assembly completeness and continuousness. However, with sufficient SRs, assemblies on the output of ECTools and pacBioToCA could achieve genomic fractions close to 1. To date, a handful of algorithms have been developed to assemble genomes with built-in error correction modules [24, 46, 47].

### Performance on correcting bases at heterozygous positions

Given that heterozygosity study is a crucial for diploid or polyploid organisms, we assessed the performance of TGS error correction methods on correcting bases at heterozygous positions of simulated human genomic data: paternal and maternal genomic LRs were corrected by randomly mixed SRs from these two haplotypes. We presented the results of FMLRC and LoRDEC here, as they were time- and memory-efficient to handle the human data of large volume (Additional file 2: Note 3, Additional file 10: Table S8).

Many errors at heterozygous positions of the simulated PacBio LRs remained uncorrected (6.19–12.36% false negative rate (FNR) in our tests), but also a number of correct bases at heterozygous positions were altered to errors by FMLRC or LoRDEC (5.66–13.32% false positive rate (FPR) in our tests). Both FPR and FNR of FMLRC increased substantially with SR coverage from 5× to 20×: FPR rose from 5.66 to 9.50% and FNR from 6.19 to 9.49%. On the contrary, FPR and FNR of LoRDEC decreased from 13.32 to 5.96% and from 12.36 to 9.13%, respectively. That is, shallow SR coverage (e.g., 5×) is relatively beneficial for FMLRC to maintaining information of haplotypes during correction whereas higher SR coverage (e.g., 20×) is favorable by LoRDEC.

FPR and FNR were higher for the simulated ONT LRs than those for the corresponding PacBio LRs (e.g., FPR: 9.51% vs. 5.66% by FMLRC with 5× SRs). It indicates that it is more challenging to maintain information of haplotypes when correcting ONT data. As SR coverage increased from 5× to 20×, FPR and FNR for the simulated ONT data corrected by FMLRC rose dramatically (from 9.51 to 14.26% and from 9.75 to 14.44%, respectively) as for the simulated PacBio data, while there was only mild difference for correcting the simulated ONT data by LoRDEC (from 15.91 to 14.77% and from 16.01 to 15.35%, respectively).

Although FMLRC and LoRDEC showed superior performance in the other evaluations, neither of them could recover/maintain true heterozygous bases in PacBio or ONT LRs with low FPR or FNR. Therefore, error correction on heterozygous genomic LRs remains a tough problem, as shown in SGS genomic data previously [48].

### Comparison between self correction and hybrid correction

Both self correction and hybrid correction can increase accuracy of LRs (Additional file 1: Figure S14, Additional file 2: Note 4). Pass number is the number of raw reads (e.g., subreads) to generate corrected consensus (e.g., CCS reads). The accuracy of CCS reads with at least two passes was generally higher than subreads (median 0.9069–0.9961 vs. 0.8728), which increased as pass number increased and saturated at pass number of 5. Similarly, ONT 2D reads also generally had higher accuracy than the raw reads (named template reads) (median 0.8572 vs. 0.6807). It should be noted that the accuracy of 2D LRs was not high, because the accuracy of the raw ONT data is low and the maximal pass number for ONT data is only 2.

Using hybrid correction, PacBio reads reached a saturated accuracy with ≥ 50× SRs (median 1.0000), which was comparable to the saturated accuracy of PacBio CCS reads with ≥ 5 passes (median 0.9961). The accuracy of ONT 2D reads (median 0.8572) was generally between hybrid correction with 5× SR coverage (median 0.6867) and 20× SR coverage (median 0.9526), while it was far lower than the saturated accuracy with ≥ 50× SRs (median 1.0000). Considering the trade-off between read length and pass number of PacBio CCS reads [1], and the relatively low accuracy of ONT 2D reads (median < 0.9), hybrid correction could be very helpful to obtain highly accurate LRs.

## Discussions and future directions

Given the strengths of LR length and no requirement on PCR, use of TGS technologies will inevitably continue to expand [1]. Since the corresponding high error rate is inherent to the low signal-to-noise ratio in single-molecule sequencing technologies, it will be hard to achieve the accuracy of SGS in the near future [39]. Thus, error correction prior to downstream data analyses is essential. Self correction and hybrid correction both can increase accuracy of raw LRs, while self correction has remarkable limits: the greater number of passes can increase accuracy of PacBio CCS reads yet at the cost of read length, and the highest pass number of ONT data is only 2. Hybrid correction could be helpful to obtain highly accurate LRs. Furthermore, hybrid error correction using SRs can rescue LRs so that they can be alignable and usable for assembly, which can reduce the relatively high cost of TGS data [32].

In this study, we assessed ten existing error correction methods in terms of sensitivity, accuracy, output rate, alignment rate, output read length, run time, and memory usage. The test data included four PacBio datasets as well as two ONT datasets from four model organisms with five different SR coverages. Overall, the graph-based methods compared favorably with the alignment-based methods. FMLRC in particular had slightly better overall performance than the other graph-based methods. However, if only low SR coverage data are available, the graph-based methods are not as robust as the alignment-based methods. The alignment-based methods except the selective methods generally have comparable yet slightly lower sensitivity, accuracy, output rate, and alignment rate, while the primary drawbacks of the alignment-based methods are long run time and high memory usage. ECTools and pacBioToCA require similar memory usage as the graph-based methods. Although it is not clear how and what criteria Jabba, ECTools, and pacBioToCA use to select and trim reads, these methods may still satisfy some specific research interests, such as scenarios that require very high accuracy but are not concerned with read length or data loss.

Unlike SR coverage, LR coverage is not a key factor affecting performance of hybrid-correction methods, because each LR is treated independently in hybrid correction regardless of the types of algorithms. Some procedures that rely on LR coverage, such as LR layout generation or consensus inference, are rarely carried out in hybrid correction, yet the LR coverage needs to be taken into account in self-correction methods [26].

Generally, the tested methods had better overall performance on the small datasets than the large datasets. Theoretically, both the alignment-based and the graph-based algorithms corrected LRs separately, so the relatively poorer performance on the large datasets is likely due to the complexity of the genomes of *D. melanogaster* and *A. thaliana*. de Bruijn graphs from complex genomes could contain more branches, bubbles, or other complicated structures [49]. On the other hand, in a complex genome, the alignment of SRs against LRs could also include a number of false positive results [28]. In particular, high repeat content across a genome poses substantial challenges to error correction, such as the stage of assigning SRs to LRs in alignment-based methods, as well as the process of searching LR-matched paths in de Bruijn graphs constructed with SRs in graph-based methods [38, 50, 51].

In addition, parameter setting is an important consideration to run the error correction methods. For example, the choice of *k*-mer length is key for the performance of the graph-based and the dual-based methods. For example, Jabba was designed to correct LRs by de Bruijn graph with a relatively large *k*-mer size (e.g., 71 or 75), which aimed to gain high accuracy per base as well as to resolve repeats in the graph [33]. On the contrary, LoRDEC was developed with short *k*-mer (e.g., 17 or 19 for data from small genomes, and 21 for large genomes). The coverage threshold of *k*-mers is another important parameter in LoRDEC: lower values result in more complex graphs and higher values cause gaps of de Bruijn graph. Besides, the number of threads affects the run time: shorter run times with more threads. The parameter setting details in our work is included in Additional file 2: Note 1.

In addition to the abovementioned quantitative metrics, the difficulty of installation and software implementation cannot be quantified but is also a critical issue for the practical application of these methods (see the corresponding details of ten methods in Additional file 2: Note 5 “Installation and implementation details,” Additional file 11: Table S9). The most common issues include the need to (1) install two to three other software such as aligners and graph constructors, and often with specific versions and (2) pre-process data, such as building an index of SRs and assembly of SRs. Any failure of these steps halts the error correction. For example, FMLRC had the best output when sufficient SR coverage is applied, while users must build a BWT index from SRs using the software ropebwt2 and msbwt. When the index builder runs incorrectly or fails, an incorrect index file with a similar file size to the correct index can still be output. FMLRC can run successfully with this incorrect index without a clear warning or error message, yet the output would have even worse quality than the input. Albeit only a single command line is needed to run LoRDEC, its installation may pose considerable challenges: each version of LoRDEC may require the installation of GATB core with a specific version, and GATB core installation is particularly problematic. The installation for Nanocorr is very complicated, requiring BLAST and its implementation is recommended with Sun Grid Engine or a similar scheduler. CoLoRMap and LSC are comparably more user-friendly, which only need a single command line to run. The ideal and new methods should include simple modules that automatically confirm the versions and installation of the required software as well as integrate the pre-processing of data with error correction in a single command line.

According to different research interests, users may place different weight on each of the performance metrics. For example, high accuracy (e.g., > 0.99) is important for studies requiring single nucleotide resolution (e.g., detection of alternative splice sites in transcriptome research and breakpoints in cancer research) as well as sequence analysis (e.g., open reading frame analysis) [52]. In the other areas, such as detection of gene isoforms, fusion genes and abundance estimation, a high alignment rate would be very useful even with relatively low accuracy (e.g., 0.90–0.95) [41]. Therefore, users should always have a comprehensive consideration of data size, computing resources, and research interests when selecting a method for error correction. On the other hand, our performance evaluation identifies specific factors that can be improved in future optimization of existing error correction methods or the development of new methods.

## Additional files

Additional file 1: **Figure S1.** Improvements in TGS will lead to further adoption. **Figure S2.** Performance plots on sensitivity of ten methods on ONT datasets using five SR coverages. **Figure S3.** Performance plots on accuracy of ten methods on ONT datasets using five SR coverages. **Figure S4.** Performance plots on output rate of ten methods on PacBio (a) and ONT (b) datasets using five SR coverages. **Figure S5.** Output rates and alignment rates of ten methods on ONT datasets using five SR coverages. **Figure S6.** Performance plots on alignment rate of error correction methods using five SR coverages on PacBio small (a) and large datasets (b), as well as ONT datasets (c). **Figure S7.** Output lengths of correction methods on *S. cerevisae* (a), *D. melanogaster* (b) and *A. thaliana* (c) PacBio datasets using five SR coverages. **Figure S8.** Output lengths of correction methods on *E. coli* (a) and *S. cerevisae* (b) ONT datasets using five SR coverages. **Figure S9.** Performance plots on run time of ten methods on PacBio (a) and ONT (b) datasets using five SR coverages. **Figure S10.** Run time and memory usage of ten methods on ONT datasets using five SR coverages. **Figure S11.** Performance plots on memory usage of ten methods on PacBio (a) and ONT (b) datasets using five SR coverages. **Figure S12.** Radar charts of correction methods on *S. cerevisae* (a), *D. melanogaster* (b) and *A. thaliana* (c) PacBio datasets using five SR coverages. **Figure S13.** Radar charts of correction methods on *E. coli* (a) and *S. cerevisae* (b) ONT datasets using five SR coverages. **Figure S14.** Comparison between self correction and hybrid correction on *E. coli* data in terms of accuracy. (PDF 940 kb)

Additional file 2: **Note 1.** Parameter settings of error correction methods. **Note 2.** Parameter settings of error correction methods. **Note 3.** Performance evaluation details on correcting heterozygous genomic data. **Note 4.** Comparison details between self correction and hybrid correction on PacBio and ONT data. **Note 5.** Installation and implementation details. (PDF 358 kb)

Additional file 3: **Table S1.** Performance statistics on sensitivity. a. Sensitivity performance statistics on PacBio data of ten methods using five SR coverages. b. Sensitivity performance statistics on ONT data of ten methods using five SR coverages. (XLSX 20 kb)

Additional file 4: **Table S2.** Performance statistics on accuracy. a. Accuracy performance statistics on PacBio data of ten methods using five SR coverages. b. Accuracy performance statistics on ONT data of ten methods using five SR coverages. (XLSX 22 kb)

Additional file 5: **Table S3.** Performance statistics on output rate. a. Output rate (%) performance statistics on PacBio data of ten methods using five SR coverages. b. Output rate (%) performance statistics on ONT data of ten methods using five SR coverages. (XLSX 19 kb)

Additional file 6: **Table S4.** Performance statistics on alignment rate. a. Alignment rate (%) performance statistics on PacBio data of ten methods using five SR coverages. b. Alignment rate (%) performance statistics on ONT data of ten methods using five SR coverages. (XLSX 22 kb)

Additional file 7: **Table S5.** Performance statistics on run time. a. Run time performance statistics on PacBio data of ten methods using five SR coverages (Unit: second). b. Run time performance statistics on ONT data of ten methods using five SR coverages (Unit: second). (XLSX 19 kb)

Additional file 8: **Table S6.** Performance statistics on memory usage. a. Memory usage performance statistics on PacBio data of ten methods using five SR coverages (unit: G). b. Memory usage performance statistics on ONT data of ten methods using five SR coverages (unit: G). (XLSX 17 kb)

Additional file 9: **Table S7.** Performance statistics on improving de novo assembly. a. Genome assembly performance statistics of ten methods on *E. coli* PacBio dataset using five SR coverages. b. Genome assembly performance statistics of ten methods on *S. cerevisae* PacBio datasets using five SR coverages. c. Genome assembly performance statistics of five methods on *D. melanogaster* PacBio dataset using five SR coverages. d. Genome assembly performance statistics of five methods on *A. thaliana* PacBio dataset using five SR coverages. e. Genome assembly performance statistics of ten methods on *E. coli* ONT dataset using five SR coverages. f. Genome assembly performance statistics of ten methods on *S. cerevisae* ONT dataset using five SR coverages. (XLSX 48 kb)

Additional file 10: **Table S8.** Performance of error correction methods on correcting bases at heterozygous positions. (XLSX 15 kb)

Additional file 11: **Table S9.** Memory usage performance statistics by the preprocessing modules of five error correction methods (unit: G). (XLSX 14 kb)
