# Supplementary material for: A comparative evaluation of hybrid error correction methods for error-prone long reads
Source: Genome Biol. 2019 Feb 4;20:26. doi: 10.1186/s13059-018-1605-z (PMC6362602; doi:10.1186/s13059-018-1605-z)
Supplement: Supplementary file 1 — Figure S1. Improvements in TGS will lead to further adoption. Figure S2. Performance plots on sensitivity of ten methods on ONT datasets using five SR coverages. Figure S3. Performance plots on accuracy of ten methods on ONT datasets using five SR coverages. Figure S4. Performance plots on output rate of ten methods on PacBio (a) and ONT (b) datasets using five SR coverages. Figure S5. Output rates and alignment rates of ten methods on ONT datasets using five SR coverages. Figure S6. Performance plots on alignment rate of error correction methods using five SR coverages on PacBio small (a) and large datasets (b), as well as ONT datasets (c). Figure S7. Output lengths of correction methods on S. cerevisae (a), D. melanogaster (b) and A. thaliana (c) PacBio datasets using five SR coverages. Figure S8. Output lengths of correction methods on E. coli (a) and S. cerevisae (b) ONT datasets using five SR coverages. Figure S9. Performance plots on run time of ten methods on PacBio (a) and ONT (b) datasets using five SR coverages. Figure S10. Run time and memory usage of ten methods on ONT datasets using five SR coverages. Figure S11. Performance plots on memory usage of ten methods on PacBio (a) and ONT (b) datasets using five SR coverages. Figure S12. Radar charts of correction methods on S. cerevisae (a), D. melanogaster (b) and A. thaliana (c) PacBio datasets using five SR coverages. Figure S13. Radar charts of correction methods on E. coli (a) and S. cerevisae (b) ONT datasets using five SR coverages. Figure S14. Comparison between self correction and hybrid correction on E. coli data in terms of accuracy. (PDF 940 kb) [file 13059_2018_1605_MOESM1_ESM.pdf]

Supplementary figures

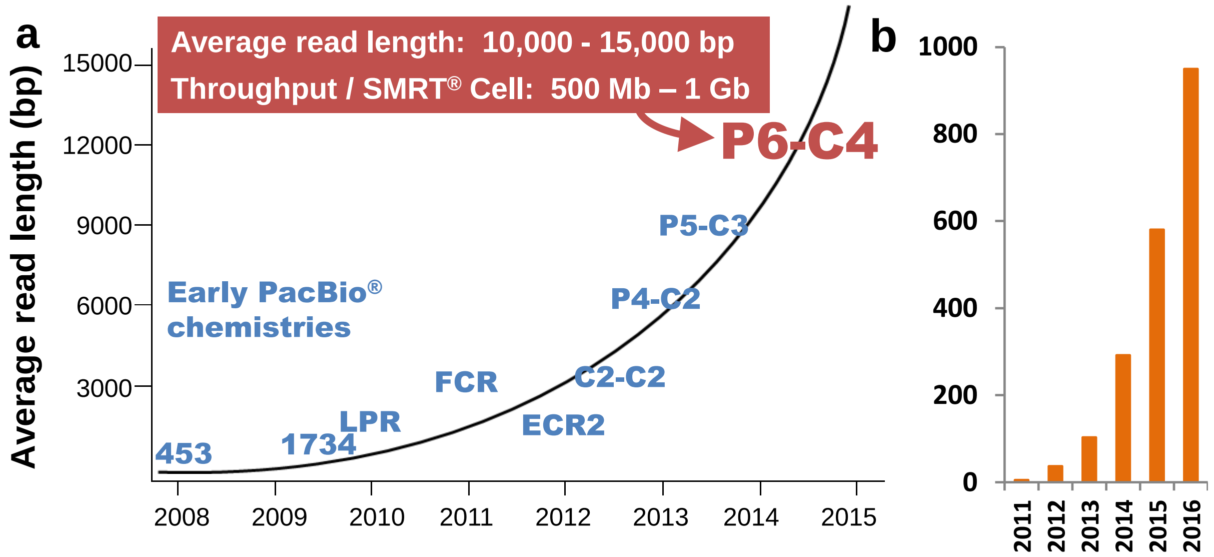

Fig S1. Improvements in TGS will lead to further adoption. (a) New PacBio sequencing chemistry is improving the data quality. **(b)** Exponential increase in the cumulative publications using PacBio data. Note: the figures were made from the data provided by PacBio company.

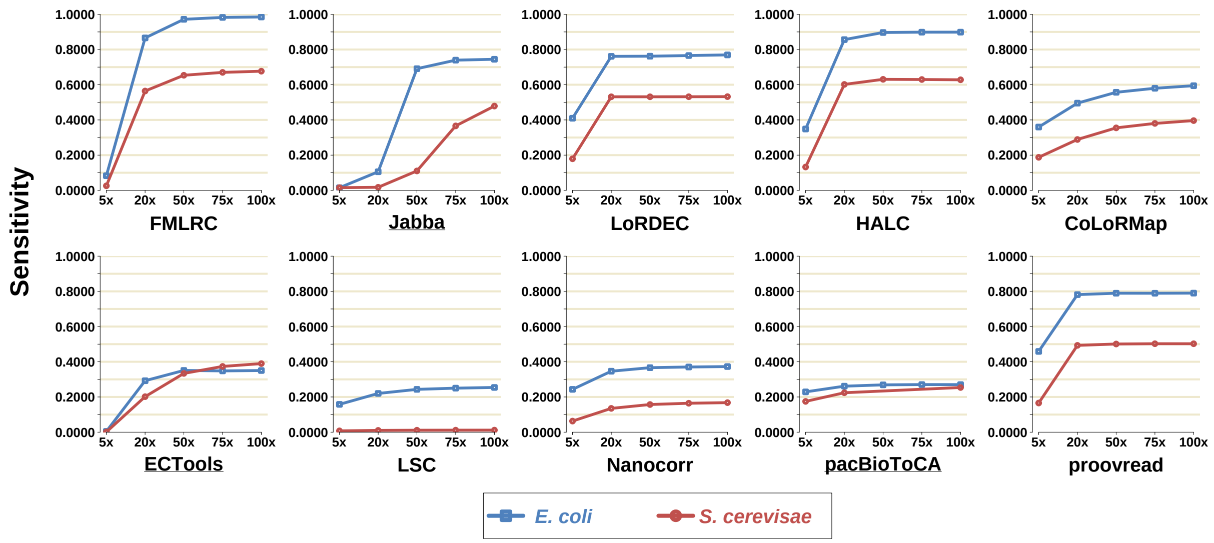

Fig S2. Performance plots on sensitivity of ten methods on ONT datasets using five SR coverages. The notations are the same as Fig 1.

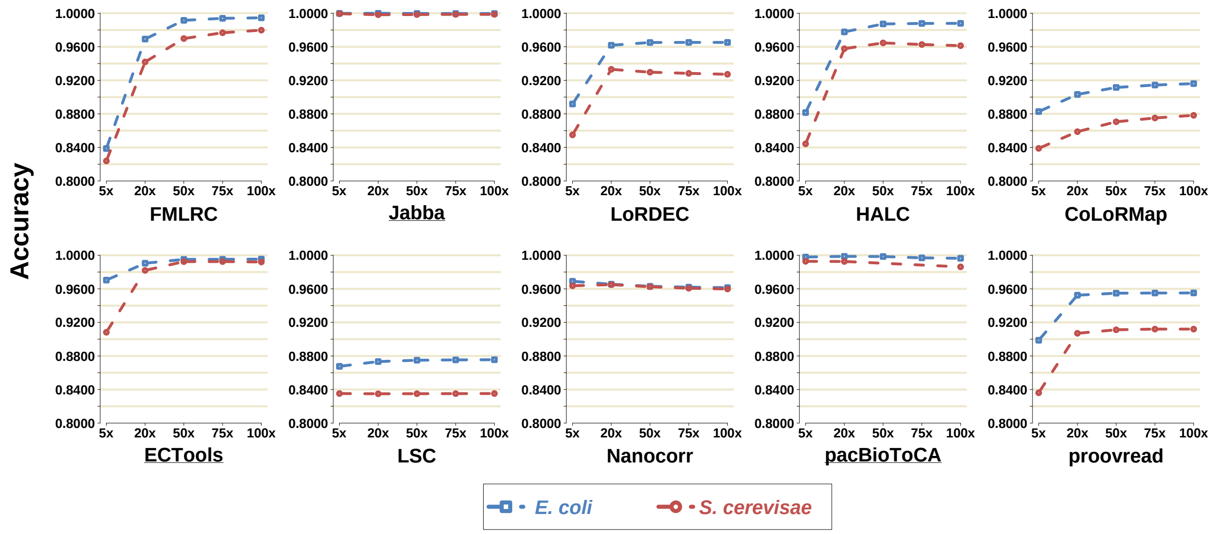

Fig S3. Performance plots on accuracy of ten methods on ONT datasets using five SR coverages. The notations are the same as Fig 2.

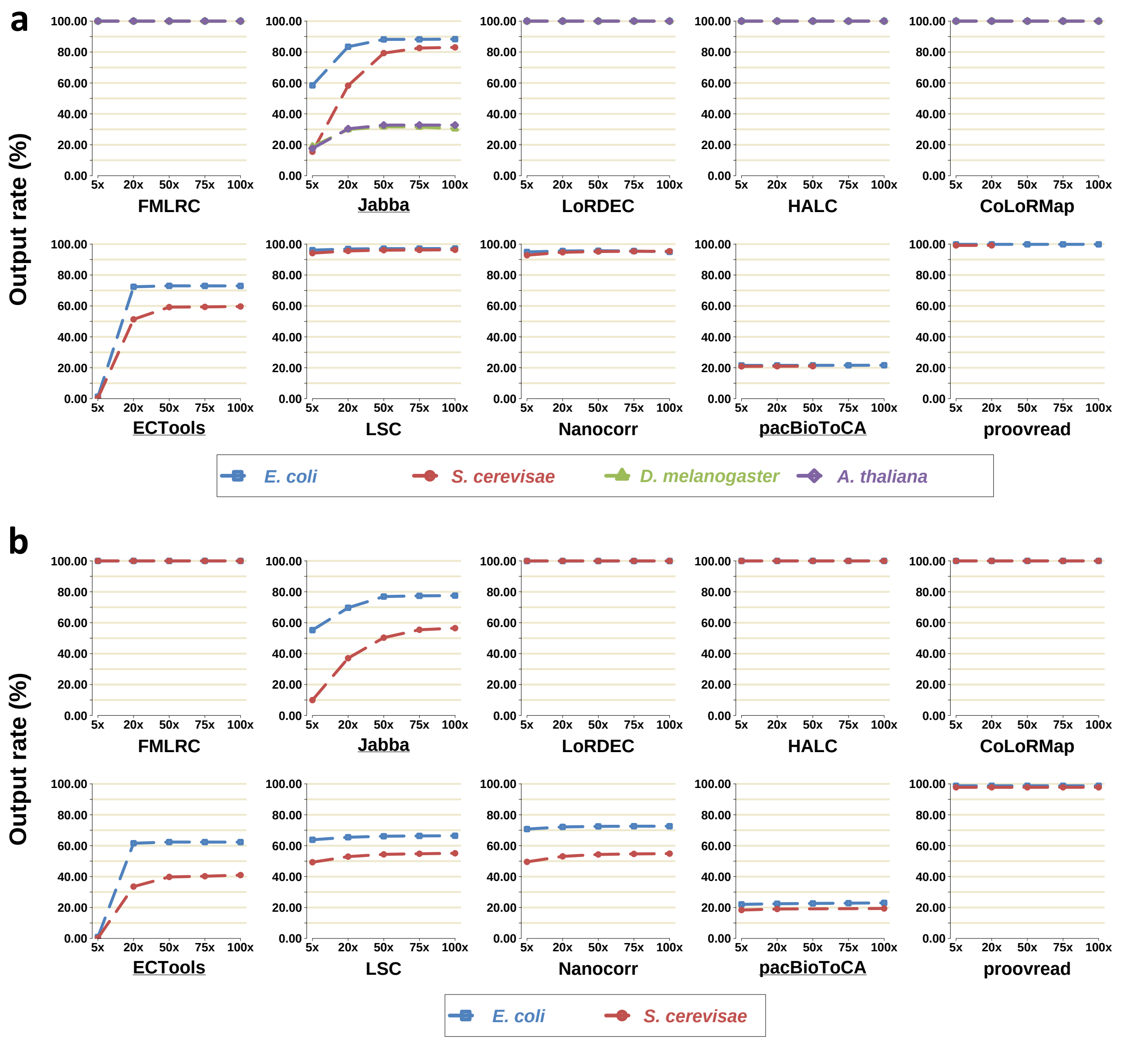

Fig S4. Performance plots on output rate of ten methods on PacBio (a) and ONT (b) datasets using five SR coverages.

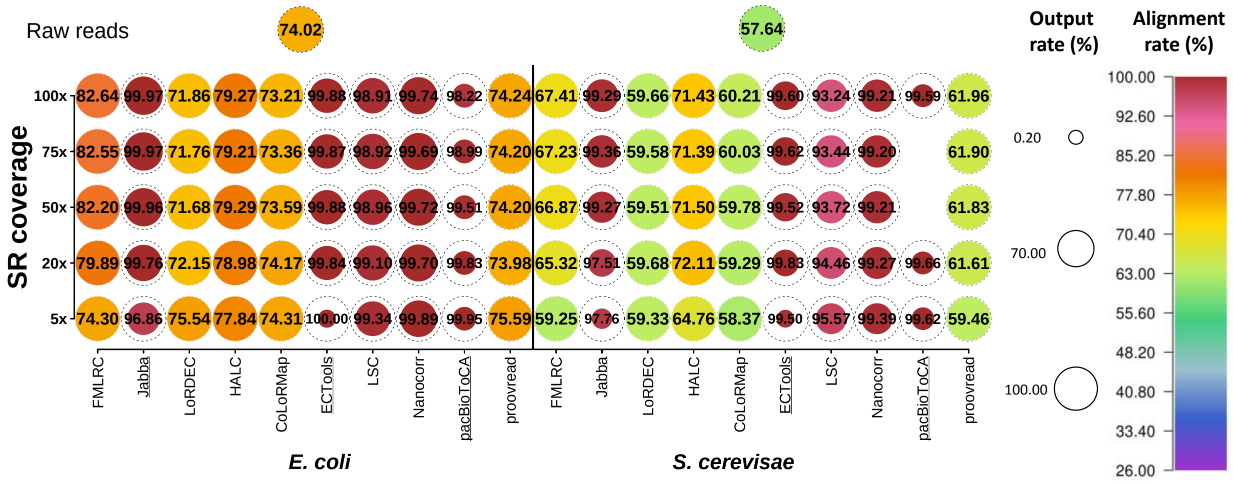

Fig S5. Output rates and alignment rates of ten methods on ONT datasets using five SR coverages. The notations are the same as Fig 3.

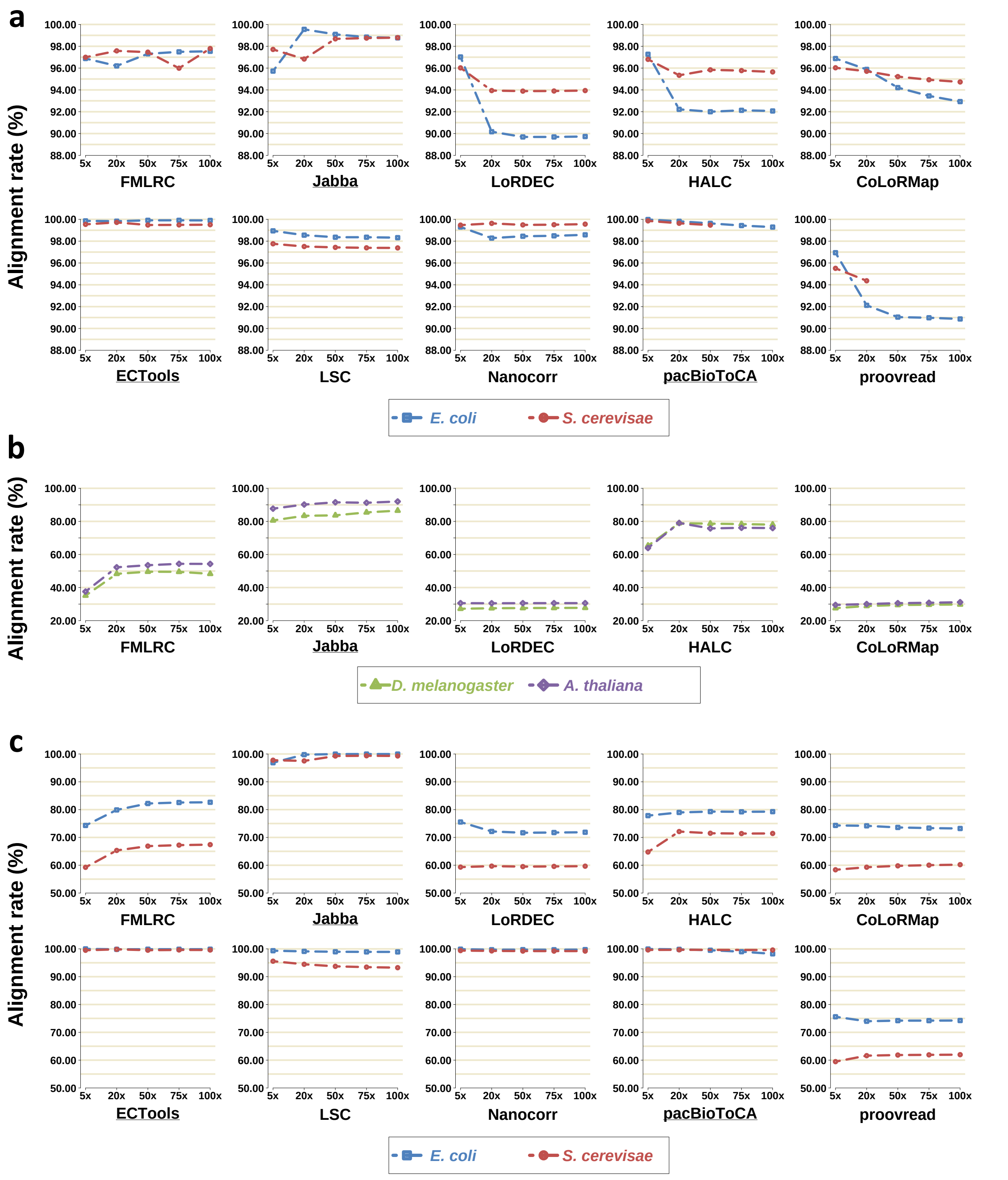

Fig S6. Performance plots on alignment rate of error correction methods using five SR coverages on PacBio small (a) and large datasets (b), as well as ONT datasets (c).

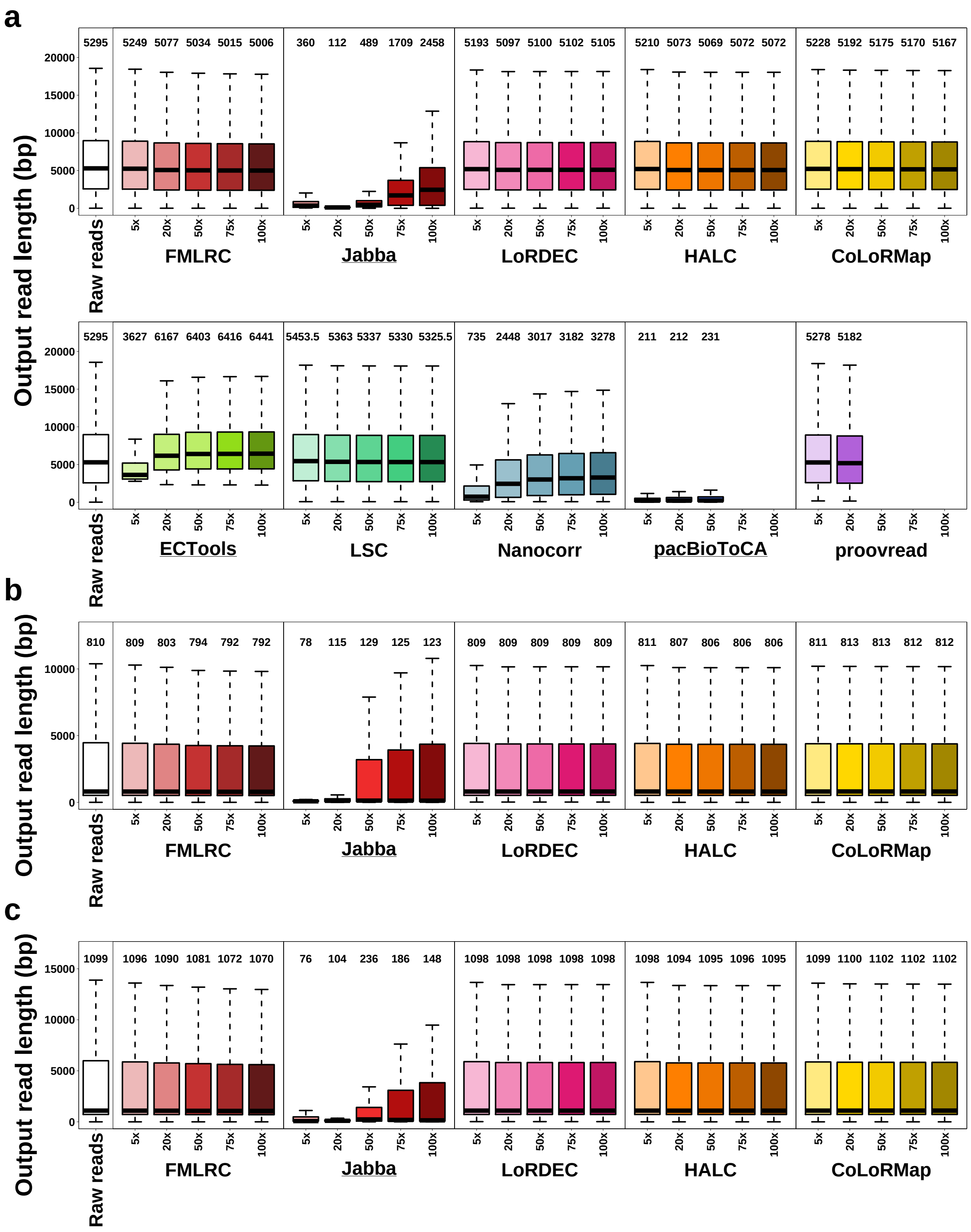

Fig S7. Output lengths of correction methods on *S. cerevisae* (a), *D. melanogaster* (b) and *A. thaliana* (c) PacBio datasets using five SR coverages. The notations are the same as Fig 5.

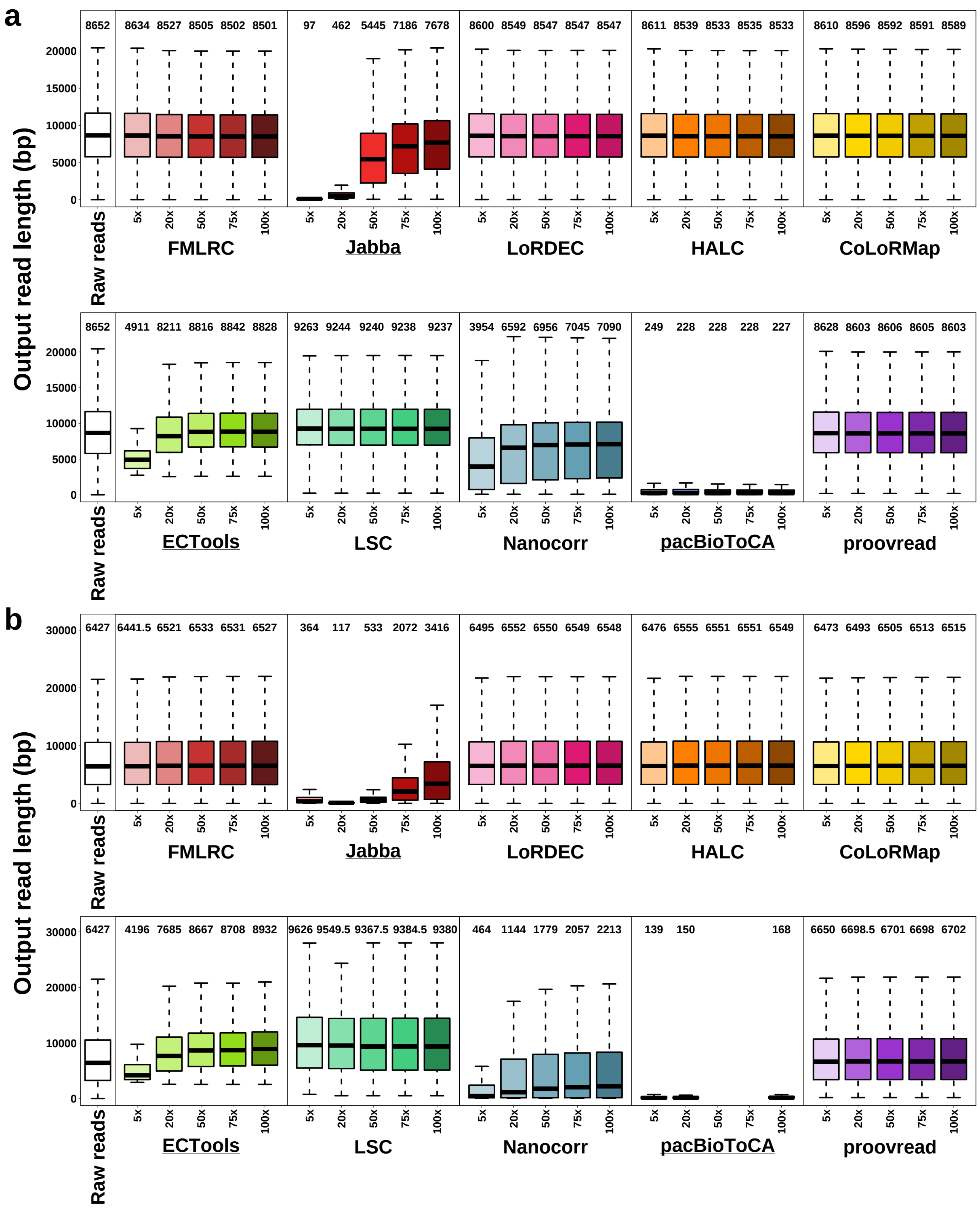

Fig S8. Output lengths of correction methods on *E. coli* (a) and *S. cerevisae* (b) ONT datasets using five SR coverages. The notations are the same as Fig 5.

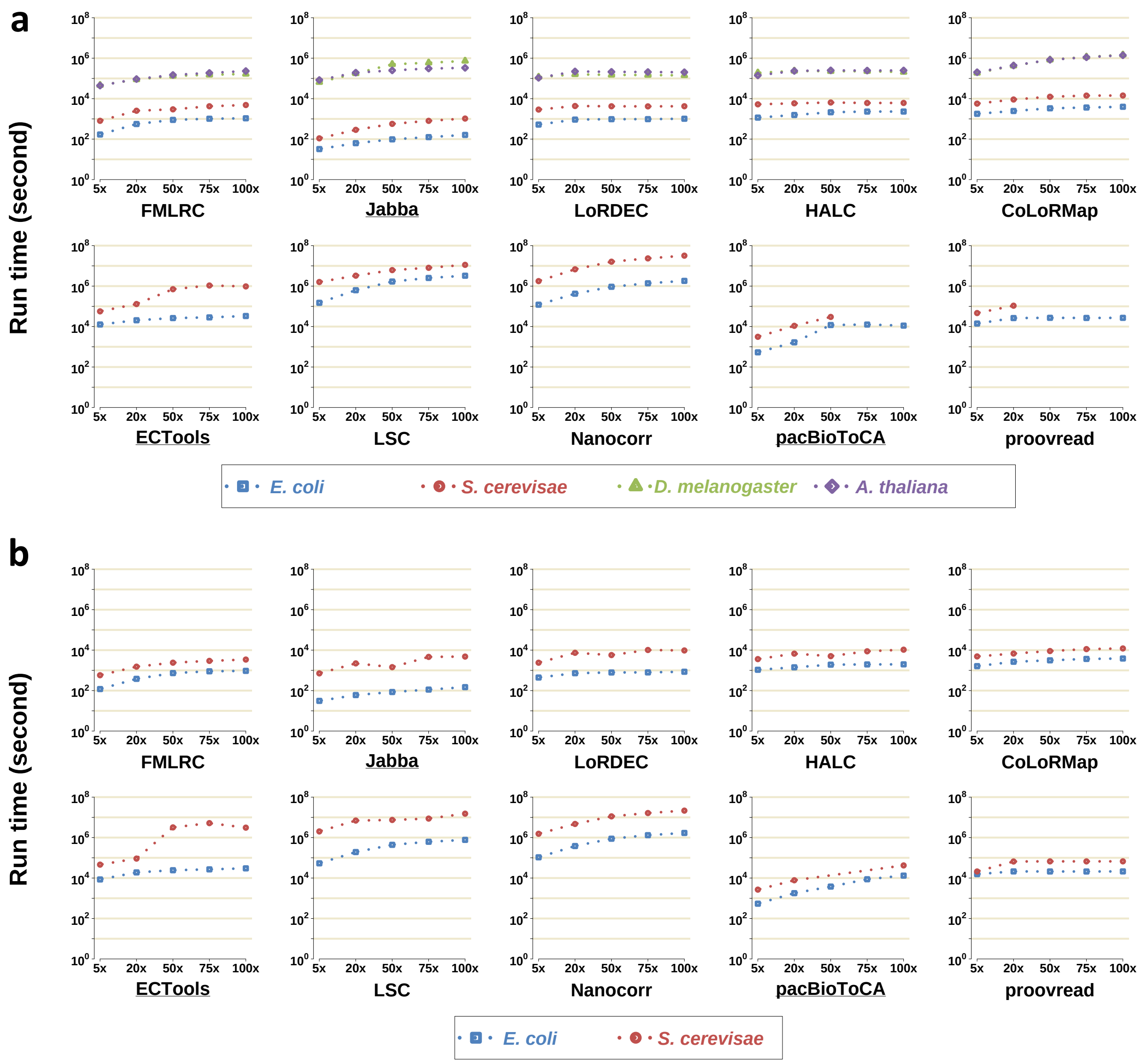

Fig S9. Performance plots on run time of ten methods on PacBio (a) and ONT (b) datasets using five SR coverages.

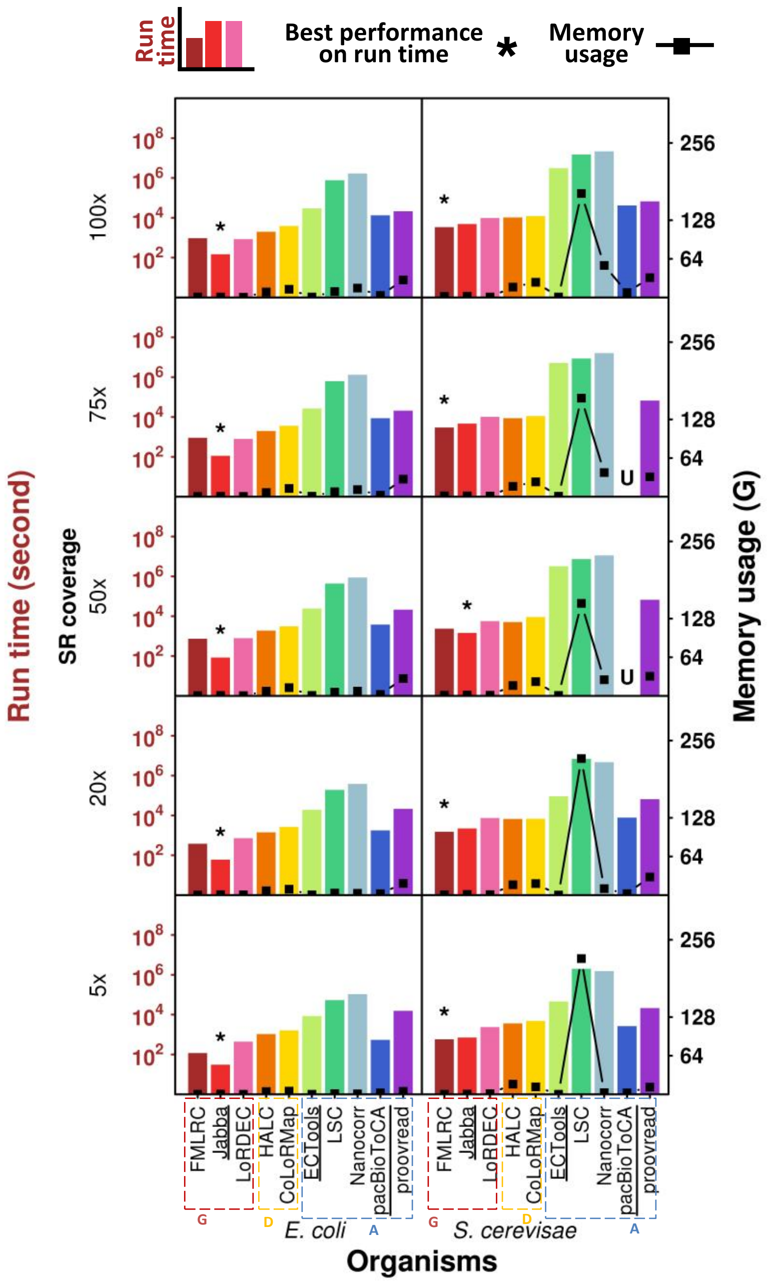

Fig S10. Run time and memory usage of ten methods on ONT datasets using five SR coverages. The comparison was performed on *E. coli* and *S. cerevisae* datasets. The performances for run times are shown with bars and for memory usages are shown with line plots. The best performance of run time is labeled with an asterisk. Methods are organized from left-to-right as follows: "G"- graph-based (FMLRC, Jabba and LoRDEC), "D"- dual-based (HALC and CoLoRMap), and "A" - alignment-based (ECTools, LSC, Nanocorr, pacBioToCA and proovread). "U" - the method failed due to **U**nidentified issue. LSC crashed on partial *S. cerevisae* dataset.

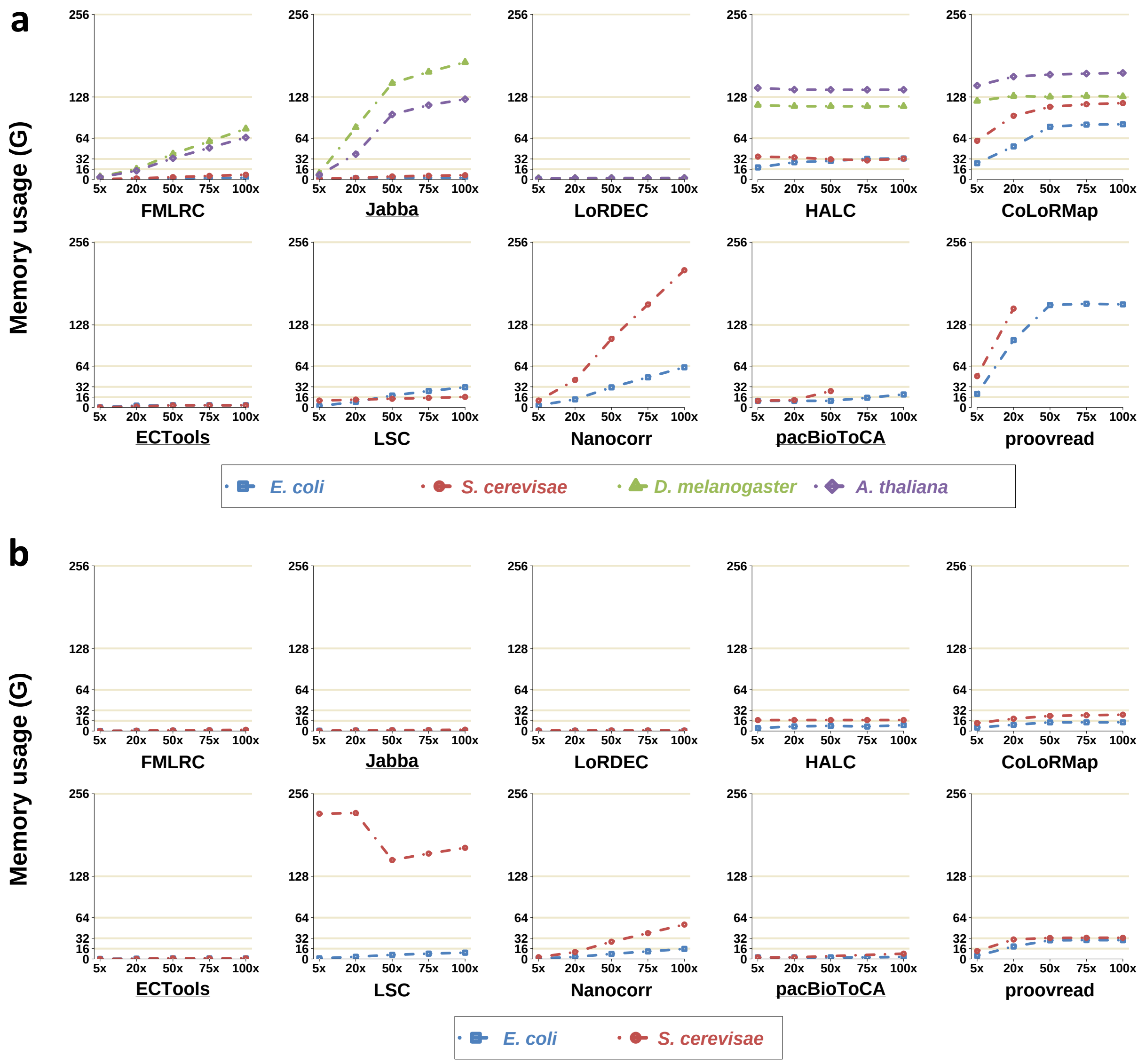

Fig S11. Performance plots on memory usage of ten methods on PacBio (a) and ONT (b) datasets using five SR coverages.

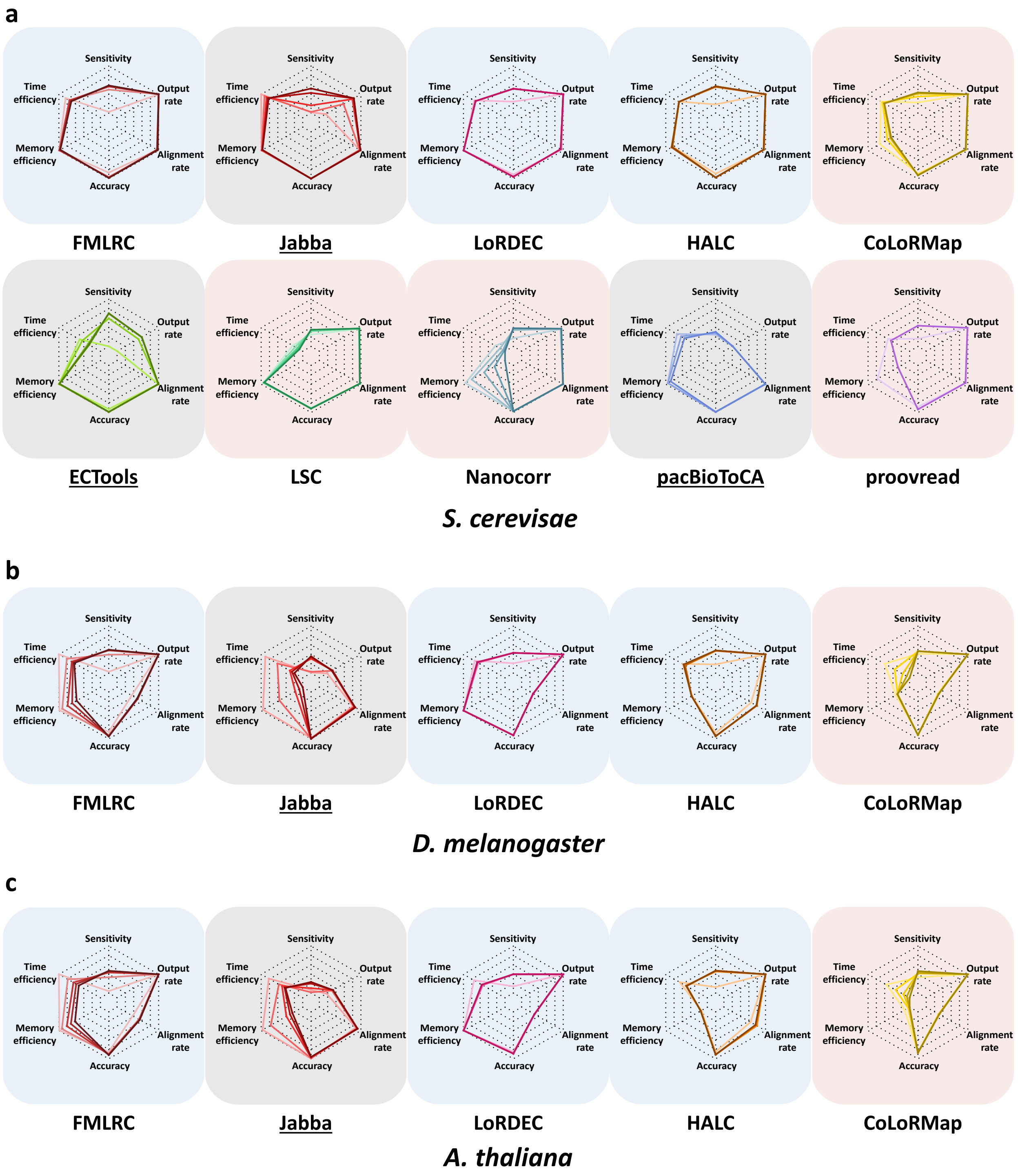

Fig S12. Radar charts of correction methods on *S. cerevisae* (a), *D. melanogaster* (b) and *A. thaliana* (c) PacBio datasets using five SR coverages. The six metrics include sensitivity, time efficiency, memory efficiency, accuracy, alignment rate and output rate. The color depth is positively correlated to SR coverage. Time efficiency is computed as the log10 transformed run time that is further normalized to 0-1 range: 0 corresponds to the longest run time and 1 corresponds to the shortest run time among tested methods with five SR coverages. Memory

efficiency is computed as the memory usage that is normalized to 0-1 range: 0 corresponds to highest memory usage and 1 corresponds to the lowest memory usage among ten methods with five SR coverages. According to the overall performance, ten methods are grouped and shaded: blue – the graph-based FMLRC and LoRDEC plus the dual-based HALC; pink – the alignment-based LSC, Nanocorr and proovread plus the dual-based CoLoRMap; gray – the selective methods Jabba, ECTools and pacBioToCA.

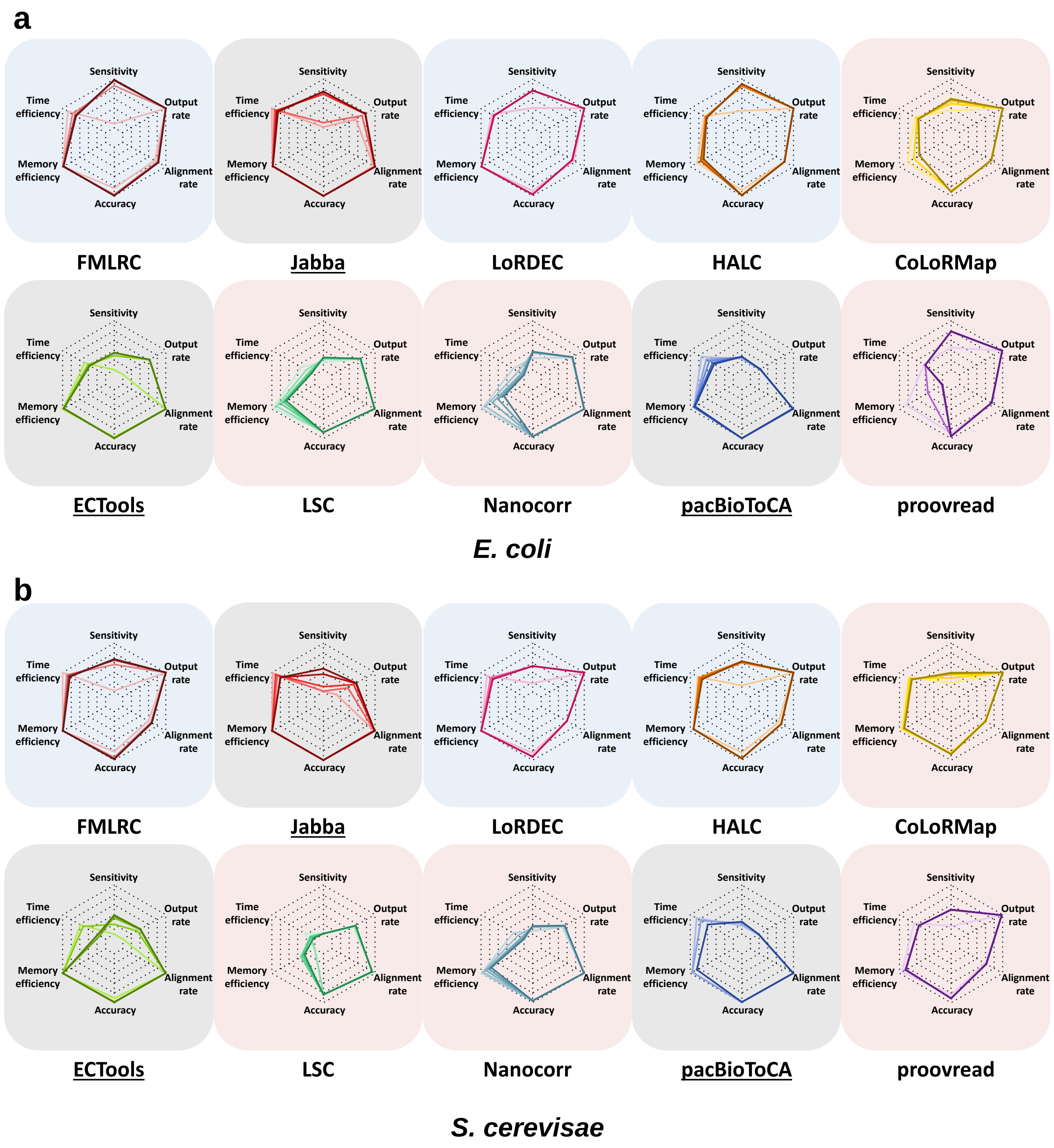

Fig S13. Radar charts of correction methods on *E. coli* (a) and *S. cerevisae* (b) ONT datasets using five SR coverages. The notations are the same as Fig S12.

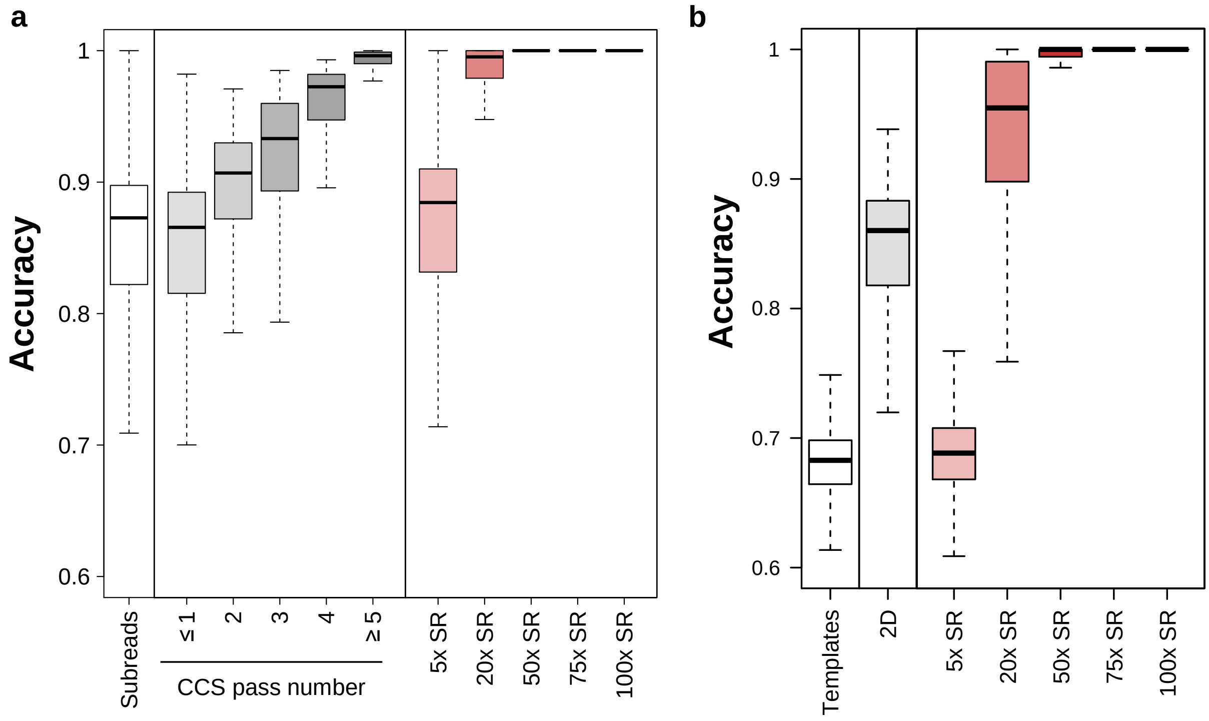

Fig S14. Comparison between self correction and hybrid correction on *E. coli* data in terms of accuracy. LRs were produced by the PacBio (**a**) or ONT (**b**) platform. Hybrid correction was performed by FMLRC. Outliers are not shown.
