# Supplementary material for: A comparative evaluation of hybrid error correction methods for error-prone long reads
Source: Genome Biol. 2019 Feb 4;20:26. doi: 10.1186/s13059-018-1605-z (PMC6362602; doi:10.1186/s13059-018-1605-z)
Supplement: Supplementary file 2 — Note 1. Parameter settings of error correction methods. Note 2. Parameter settings of error correction methods. Note 3. Performance evaluation details on correcting heterozygous genomic data. Note 4. Comparison details between self correction and hybrid correction on PacBio and ONT data. Note 5. Installation and implementation details. (PDF 358 kb) [file 13059_2018_1605_MOESM2_ESM.pdf]

Supplementary materials

Note 1: Parameter settings of error correction methods

FMLRC

A Burrows-Wheeler transform (BWT) of short reads (SRs) built with msbwt (version 0.3.0) and ropebwt2 (version r187) was used as one the inputs of FMRLC. msbwt was run with Python modules of pysam (version 0.11.2), argparse (version 1.4.0) and Numpy (version 1.10.1). The parameters were set as suggested.

Jabba

Karect (version 1.0) was used to correct errors in SRs for small datasets with parameters set as suggested, but not applied to large datasets given memory limitation. Brownie (version 1.0) was used to build de Bruijn graph, followed by Jabba for LR error correction, both with k=71.

LoRDEC

LoRDEC was run with k=19 for the small datasets with small genomes, and k=21 for large datasets with large genomes as suggested. The solid k-mer threshold was set as 3. Other parameters were set as default.

HALC

Contigs generated from SRs with SOAPdenovo2 (version 2.04), with k=25, k-mer frequency cutoff d=3 and other parameters set as suggested, were used as a part of the inputs of HALC. BLASR (version 5.3) [1] and LoRDEC (version 0.5.3) were used in HALC pipeline.

CoLoRMap

Parameters were set as default.

ECTools

Celera unitigs were generated with Celera Assembler (version 8.1), and used as a part of the inputs of ECTools. The Nucmer suite from MUMmer (version 3.23) was used in ECTools pipeline. The long reads (LRs) were partitioned into small batches with 100 each.

LSC

Bowtie2 (version 2.2.5) [2] was used as the aligner, and parameters were as default. The LRs were partitioned into small batches with 100 each.

Nanocorr

The packages Cython (version 0.26), NumPy (version 1.10.1), h5py (version 2.7.0), pbcore_python (version 2015-8-21), pbdagcon_python (version 2015-08-21), jbio (version 2015-11-11), jptools (version 2015-11-11) and aligner BLAST (version 2.6.0) were used in Nanocorr pipeline. The LRs were partitioned into small batches with 100 each as suggested.

pacBioToCA

The parameter "genomeSize" was set as the size of the corresponding reference genome, and other parameters were set as suggested.

proovread

The parameter "estimated SR coverage" was set as the coverage of SRs used, and other parameters were set as suggested.

Input LRs from large datasets for FMLRC, Jabba, LoRDEC and CoLoRMap were split into 20 batches for their implementation.

Note 2: Performance evaluation details of error correction methods on improving *de novo* assembly

We assessed the performance on improving *de novo* assembly of the correction methods on PacBio and ONT datasets using five SR coverages. We applied Miniasm [3], a *de novo* assembly approach without read correction and consensus calling [4]. Only LRs with length of at least 15 bp were used because of the input length restriction of Miniasm. The assembly metrics, including number of contigs, N50, genome fraction that the resulting assembly covers as well as contig sequence accuracy, were applied [4]. Among these assembly metrics, contig sequence accuracy (i.e. 1-error rate of contig sequences) was evaluated by alignment of resulting contigs to corresponding reference genomes by BLASR. Contig sequence error rates were calculated as the number of identical bases divided by the length of the aligned region in the corresponding genome [5]. Some assembled contigs were shred into fragments of at most 50,000 bp for the sake of mapping feasibility, as most LRs were shorter than 50,000 bp.

Note 3: Performance evaluation details on correcting heterozygous genomic data

We simulated a diploid human genome (include paternal and maternal sequences) with probability of SNPs as $1x10^{-3}$ for each site and minor allele frequency as 5% [6, 7]. Totally 314,307 heterozygous positions were simulated. With these two mock human haplotypes, we simulated LRs (PacBio LRs by SimLoRD [8] and ONT LRs by Nanosim [9]) and SRs (by ART [10]) with 20x genome coverage respectively, considering that 20x read depth is sufficient for SNP calling [11]. The simulated LRs that covered polymorphic positions were picked out for hybrid correction, resulting in 396,027 (5.25%) paternal and 395,324 (5.24%) maternal PacBio LRs, as well as 400,805 (5.07%) paternal and 401,571 (5.08%) maternal ONT LRs. The paternal and maternal SRs were pooled together to randomly pick out reads with 5x and 20x genome coverage respectively for successive analyses, with resulting percentages of paternal reads as 44.86% (5x) and 49.06% (20x).

Two time- and memory- efficient methods, FMLRC and LoRDEC, were applied to the mock SRs and LRs above to evaluate the performance of correcting heterozygous genomic data, given to their feasibility of processing huge volume of input data. Corrected LRs were aligned to their corresponding source genome resulting in alignment rates of >99.99% (PacBio) and 83.26-89.73% (ONT), and analyzed with JVarkit [12]. Only bases at heterozygous positions covered by alignment were evaluated. Each heterozygous position in alignment was examined: true positive (TP) positions were those with errors and corrected, true negative (TN) positions were those without errors and remained unchanged after correction, false positive (FP) positions were those without errors but gained errors after correction, false negative (FN) positions were those with errors and remained erroneous after correction. We computed false positive rate (FPR) as FP/(FP+TN) and false negative rate (FNR) as FN/(TP+FN).

Note 4: Comparison details between self correction and hybrid correction on PacBio and ONT data

To compare self correction and hybrid correction on PacBio data, subreads and Circular Consensus Sequence (CCS) reads from *E. coli* data (Table 2 in the main text) were extracted with SMRT Analysis (v2.3) with pass number information, and aligned to the reference genome to calculate accuracy with BLASR. A total of 84,030 subreads, 44,633 reads with ≤ 1 CCS pass, 6,845 reads with 2 CCS passes, 705 reads with 3 CCS passes, 242 reads with 4 CCS passes and 1,020 reads with ≥ 5 CCS passes reported accuracy and were used in comparison. Subreads corrected by FMLRC with 5x, 20x, 50x, 75x and 100x SRs respectively were also aligned to the reference genome to compute accuracy.

To compare self correction and hybrid correction on ONT data, *E. coli* template and 2D LRs from *E. coli* data (Table 2 in the main text) were aligned to the reference genome, and 42,840 template and 47,461 2D LRs reported accuracy and were used in comparison. Similarly, FMLRC was applied to templates using SRs with the above-mentioned five coverages and corrected template LRs were aligned to the reference genome to compute accuracy.

Note 5: Installation and implementation details

FMLRC

Before implementing FMLRC, users need to build a BWT with SRs using ropebwt2 and msbwt. Users may experience difficulty in figuring out whether generation of the index file is successful, because if it fails, it will still generate an index file with similar size to what it should be, and FMLRC will take it as a part of the inputs without a clear warning or error message, giving output probably with lower quality to input data.

Jabba

The installation of Jabba itself is user-friendly, however, its implementation recommends to correct SR errors by Karect and to build de Bruijn graph by brownie, but these two tools need to be downloaded and installed separately before implementing Jabba. We also observed that the SR error correction tool, Karect is very memory-consuming as data size increases, and hence we did not apply it to large datasets (Table S9).

LoRDEC

Albeit the implementation of LoRDEC is very user-friendly with one command line, its installation may pose considerable challenges, each version of LoRDEC may require the installation of GATB core with a specific version, and GATB core installation is particularly problematical.

HALC

HALC requires plugs of SOAP-denovo2 and BLASR. It aligned SR-contigs assembled by SOAPdenovo2 to construct a contig graph, the installation and implementation of SOAPdenovo2 is user-friendly, but we found HALC crashed on *A. thaliana* data with 5x SR coverage. The authors from HALC paper recommended us to run it with a single thread, and it turned out to be implemented successfully on the same dataset, and thereby we divided the run time by 16 to get comparable run time with 16 CPUs for that case. HALC also requires BLASR with version ≥ 5.1 and a specific version of LoRDEC, and the running commands for these two tools are imbedded in the codes of HALC.

CoLoRMap

We find it easy to install CoLoRMap, although it requires dependencies of BWA, SAMtools and Minia, because they are included in the software package, and installation can be executed with one-line command. However, it is relatively memory-consuming on large datasets compared to graph-based methods. For example, it requires >100 G memory on our *D. melanogaster* dataset with 100x SR coverage. There is no guideline for memory usage with data size.

ECTools

Before implementation of ECTools, users need to install MUMmer and Celera separately and assemble SRs by Celera Assembler. Users are recommended to partition their LRs into smaller batches (e.g., 100 LRs in each batch) for feasible implementation, and it is more favorable for computing environment with Sun Grid Engine (SGE).

LSC

LSC requires an existing aligner to align SRs to LRs after both are processed with homopolymer compression. It also requires large disk space to store intermediate files during implementation. Python 2.7 is recommended, and python with other versions may not work.

Nanocorr

The procedure for Nanocorr installation is particularly complicated. Users need to install BLAST and are recommended to implement Nanocorr with SGE or a similar scheduler. Similar to ECTools, Users are recommended to partition their LRs into smaller batches (e.g., 100 LRs in each file) for feasible implementation.

pacBioToCA

The software pacBioToCA is a module in the package of Celera Assembler. We attempted to implement its advanced version named PBcR, but it crashed on the *E. coli* dataset, and hence we stuck to the older version pacBioToCA. SRs need to be assembled beforehand by an assembly algorithm (e.g., Celera Assembler). It requires BLAST (version ≥ 2.2.24) and SAMtools (version ≥ 1.1).

proovread

Implementation of proovread requires the plugin of BLAST. It is comparatively memory-consuming and hence it was only applied to some of small datasets.

Supplementary references

1. Chaisson MJ, Tesler G: **Mapping single molecule sequencing reads using basic local alignment with successive refinement (BLASR): application and theory.** *Bmc Bioinformatics* 2012, **13**.
2. Langmead B, Salzberg SL: **Fast gapped-read alignment with Bowtie 2.** *Nature Methods* 2012, **9:**357-U354.
3. Li H: **Minimap and miniasm: fast mapping and de novo assembly for noisy long sequences.** *Bioinformatics* 2016, **32:**2103-2110.
4. Wang JR, Holt J, McMillan L, Jones CD: **FMLRC: Hybrid long read error correction using an FM-index.** *BMC Bioinformatics* 2018, **19:**50.
5. Salmela L, Rivals E: **LoRDEC: accurate and efficient long read error correction.** *Bioinformatics* 2014, **30:**3506-3514.
6. Altshuler D, Brooks LD, Chakravarti A, Collins FS, Daly MJ, Donnelly P, Gibbs RA, Belmont JW, Boudreau A, Leal SM, et al: **A haplotype map of the human genome.** *Nature* 2005, **437:**1299-1320.
7. Kao WC, Chan AH, Song YS: **ECHO: A reference-free short-read error correction algorithm.** *Genome Research* 2011, **21:**1181-1192.
8. Stocker BK, Koster J, Rahmann S: **SimLoRD: Simulation of Long Read Data.** *Bioinformatics* 2016, **32:**2704-2706.
9. Yang C, Chu J, Warren RL, Birol I: **NanoSim: nanopore sequence read simulator based on statistical characterization.** *Gigascience* 2017, **6**.
10. Huang WC, Li LP, Myers JR, Marth GT: **ART: a next-generation sequencing read simulator.** *Bioinformatics* 2012, **28:**593-594.
11. Nielsen R, Paul JS, Albrechtsen A, Song YS: **Genotype and SNP calling from next-generation sequencing data.** *Nature Reviews Genetics* 2011, **12:**443-451.
12. Pierre L: *JVarkit: java-based utilities for Bioinformatics.* 2015.
